# Patients’ experiences of video consultations: A qualitative systematic review

**DOI:** 10.1177/20552076251404513

**Published:** 2026-01-05

**Authors:** Lina Ärlebrant, Robyn Schimmer, Anette Edin-Liljegren

**Affiliations:** 1 Department of Epidemiology and Global Health, 174480 Umeå University, The Swedish Centre of Rural Health, Region Västerbotten, Storuman, Sweden; 2 Department of Epidemiology and Global Health, 174480 Umeå University, Umeå, Sweden

**Keywords:** Telemedicine, eHealth, mHealth, qualitative, systematic review

## Abstract

**Introduction:**

Video consultation (VC) became vital for improving healthcare access during COVID-19 pandemic and remains so. Despite evidence of effectiveness, concerns including technology literacy and inconsistencies in experience highlight the need for larger, patient-focused studies. While patients appreciate the convenience of VC, challenges during complex issues and patients’ preferences for in-person care persists. Synthesising qualitative studies offers insights into the fragmented understanding of patient experiences with VC. This review explores adult patients’ experiences of VC.

**Methods:**

A systematic literature search was conducted for studies published between 2011 and 2024 and reported according to the PRISMA statement. Study quality was assessed using the CASP checklist, and data were analysed through thematic synthesis. Confidence in the findings was evaluated using GRADE-CERQual.

**Results:**

In total, 3203 unique studies were retrieved; 13 were included in the final synthesis, resulting in four main themes: (1) *suitable for less complex issues when technical problems can be solved*; (2) *feeling secure, relaxed, and having mutual focus in an equitable partnership*; (3) *limitations regarding personal needs and practical help*; and (4) *increased vulnerability and lack of emotional feedback*.

**Conclusion:**

VC is experienced as ideal for managing less complex issues but is challenging for emotional topics due to technical concerns. It empowers patients by providing a neutral place for focused conversations but can create vulnerability and distance that can challenge the patient–professional relationship. Success requires technological adaptation, sufficient time during VC, and emotional support. VC should complement – not replace – traditional care, with its use determined in dialogue with patients.

## Introduction

Video consultation (VC) enables communication through real-time visual and audio connection.^1^ Information and communication technologies such as VC are believed to be an essential factor in ensuring universal health coverage,^2^ and provide opportunities to overcome geographical distances and ensure access to healthcare in areas where it is lacking.^3^ During the COVID-19 outbreak, VC played an important role in providing continuous access to necessary health services, while keeping patients and healthcare professionals safe.^4,5^ A post-pandemic analysis of the use of telehealth, which includes VC, showed that between 2019 and 2023 there was a remarkable global effort to utilise telehealth.^6^ The year 2020 might well be the turning point where digital technology became accepted as part of healthcare routines by the public as well as governments.^7^ The effectiveness of telemedicine in healthcare has been the subject of investigation for several decades. A systematic review of reviews published in 2010 reported that 21 reviews concluded telemedicine to be effective. However, it also identified 22 reviews that found the evidence to be limited and inconsistent. This divergence underscored the need for more large-scale studies and a greater emphasis on incorporating patients’ perspectives into evaluations of telemedicine services.^8^

Comparative reviews have demonstrated that both telephone and VC are as effective as face-to-face (FTF) consultations in improving clinical outcomes across primary care,^9,10^ psychotherapy,^11^ and hospital-based settings.^12^ Moreover, VC has been found to outperform telephone consultations in a variety of healthcare contexts,^13^ offering enhanced effectiveness at significantly lower costs than traditional care.^12^ Despite the extraordinary increase in use of VC, patients still prefer FTF consultations^14–16^ and VC with healthcare professionals that they have previously met in person.^17–19^ Besides that, VC is still found challenging concerning emotional and complex questions^20,21^; and have issues related to technology.^18,22^

Patient involvement in healthcare can enhance the relevance, quality, safety, and effectiveness of healthcare services globally, and there is a need for qualitative studies that explore patients’ experiences in depth.^23^ Patient preferences are central to the delivery of high-quality care, and a paucity of research focusing on patients’ experiences of virtual care has been identified.^24^ The patient perspective contributes with recognition of the most appropriate use of VC for different patient groups and clinical conditions, and can capture potential downsides.^9,25^ Patient experiences of VC suggest that patients are comfortable communicating through video, and that VC has become normalised^18^ and is seen as a convenient tool^19,20^ that is appreciated by patients as well as by healthcare professionals.^25,26^ However, contradictions within patient experiences of VC suggest that this is a fragmented field of research, and that patients’ preferences should be better examined^25^ in order to explore what works well for different patient groups and why.^27^ Evaluation of satisfaction with VC is most often undertaken using questionnaires,^26,28^ and several studies report patient experiences based on healthcare professionals’ observations.^25^ The lack of comparable results and qualitative studies focusing on patient perspectives regarding VC highlights the need to gather existing knowledge generated by qualitative studies.

Reviews from 2019 and 2020 state that experiences, such as satisfaction, of VC have rarely been systematically reviewed using robust methodology.^14,26^ The lack of qualitative systematic reviews on patient experiences of VCs still remains since PROSPERO,^29^ an international systematic review registry, shows only two existing or ongoing reviews in this area, both focusing on a much broader field of digital healthcare solutions and telemedicine for conditions such as somatic diseases^30^ and chronic disease management during COVID-19.^31^

To enhance the delivery of digital health services such as VC, understanding the patient perspective is of unequivocal importance. While previous research has explored patients’ experiences of VC across various clinical specialisations, to our knowledge, no systematic review has yet examined the experiences between patients and healthcare professionals via VC without categorising participants by specific conditions or specialties. Despite the rapid expansion of research on VC in recent years, there remains a notable gap in studies that synthesise qualitative data to complement the insights derived from quantitative approaches. A systematic review focusing broadly on patient experiences of VC, investigated through qualitative methodologies, could offer critical insights for healthcare practitioners, telehealth system designers, and policymakers alike.

### Aim

The aim of the study was to compile and synthesise the scientific literature on adult patients’ experiences of VC with healthcare professionals.

## Methods

### Search strategy

A systematic search was undertaken in PubMed, CINAHL, MEDLINE, PsycINFO and Web of Science to find articles on patient experiences of VC in the context of healthcare. This review is reported according to the PRISMA statement guidelines,^32^ in line with the ‘Enhancing transparency in reporting the synthesis of qualitative research’ (ENTREQ) statement,^33^ and is registered in the International prospective register of systematic reviews (PROSPERO; Registration ID: CRD42022330476). The search strategy was formulated using the SPICE Framework, suitable when assessing experiences.^34^ The concepts were set as follows: Setting (S) − Healthcare; Population (P) − Patients; Intervention (I) − VC; Comparison (C) − not relevant to the review question; and Evaluation (E) − Experiences. Key terms related to the SPICE concepts was identified by test searches, a hand review of reference lists from relevant studies, and searches for synonyms and related terms using the Swedish MeSH database,^35^ supplemented with appropriate terminology sourced from Merriam-Webster's online dictionary.^36^ This approach ensured both linguistic and conceptual breadth in capturing relevant literature. As the thesaurus and subject headings used in PsycINFO differ from the more harmonised vocabularies found in Cinahl and PubMed, a separate search was conducted in PsycINFO to identify terms equivalent to those used in the search strings of the other databases. Some of the identified key terms were, telemedicine, remote consultation, mhealth, VC, audiovisual, videoconference, patient satisfaction, attitude, and perception. Further construction of the search strategy was then structured around conceptual blocks. Words categorised under the same block was combined using the Boolean operator “OR”, and blocks were combined using “AND”. The combination of the three blocks; #1 key terms related to telemedicine; #2 key terms related to VC; and #3 key terms related to patient experience, resulted in the most relevant hits and was kept for the final search. The search strategy employed a combination of controlled vocabulary terms (e.g. MeSH terms) and free-text keywords, structured using Boolean operators (AND, OR) and field-specific tags (e.g. [Mesh], [tiab], [All fields]). Truncation symbols (e.g. *) were used to capture variations of root words. Each database's specific indexing system was considered. For full details of the search strategy in the different databases, see Appendix 1, and for search strings, limitations set, and number of hits, see Appendix 2.

A significant effort was made to have a broad yet manageable search strategy. The strategy and terms were agreed upon with an information specialist.

### Literature search and study characteristics

The initial search was conducted on 11 October 2021, and complemented with a second search on 29 January 2024. Duplicates were removed from the results of the initial search using the EndNote software, and from the results of the second search using Rayyan.^37^ The remaining duplicates were identified and manually removed by the authors.

The authors co-operated on the process of study selection. Title, abstract and full-text screening was performed by two of the authors independently to include studies that fit the inclusion criteria (Table 1). Disagreement at all stages was solved through discussions between the authors; when needed, articles were reviewed by a third author.

**Table 1.** Inclusion and exclusion criteria.

	Inclusion criteria	Exclusion criteria
**Setting**	Healthcare at any level and speciality	Not healthcare, e.g. education, industry
**Population**	Adult patients > 18 years old The only active party in the conversation on the patient side of the VC	Patients < 18 years old Signs of other active parties, such as parent, partner, or next of kin, in the conversation during the VC
**Intervention**	VC, i.e. real-time audio and video communication	Recorded video instructions Text chat Phone call
**Evaluation**	Experiences Qualitative data	Mixed methods Quantitative data
**Language**	English	Not English (important due to the need to conduct the review within a reasonable timeframe)
**Year of publication**	2011 – January 2024	Papers published pre-2011, found during the test search, showed that publications in this area increased after this year
**Type of publication**	Peer-reviewed original research papers	Grey literature Dissertations and theses Conference presentations

### Quality assessment

The quality of the included studies was assessed by the authors, with two authors independently, using the Critical Appraisal Skills Programme (CASP) checklist.^38^ During the process of quality assessment from the initial search, the authors decided to exclude studies that used mixed methods, since the reporting especially regarding the recruitment strategy, the qualitative data analysis, and result section was substandard. There was also lacking information about the different steps of the analysis; references to methods used; and citations to strengthen the results; which altogether made it impossible to assess the quality in all mixed-method studies that were evaluated. The proportion of mixed-method studies included in the quality assessment was 5.4% in the initial search, and during the second search, mixed-method studies were excluded during the title, abstract and full-text screening.

### Data extraction

Data that aligned with the aim of this systematic review were extracted from the Results/Findings section of each of the studies; this included both quotations from patients, and analysis conducted by authors. To ensure a similar procedure between all the authors, all three authors extracted data from one of the studies and discussed the results to reach consensus. Data extraction was thereafter undertaken independently by the authors, and the data was imported into MAXQDA (Version: MAXQDA 2022 (Release 22.8.0)) and Excel (version 2407). The extracted data also included authors; year of publication; title; publication; country; study population; number of participants; data-collection method; data-analysis method; and research questions/aim. These are presented in Table 2.

**Table 2.** The included studies.

Author and year	Title	Publication	Country	Population	Number of participants	Data-collection method	Data-analysis method	Research questions/aim
Andrews et al., 2023	Video Chat Therapist Assistance in an Adaptive Digital Intervention for Anxiety and Depression: Reflections From Participants and Therapists	Professional Psychology: Research and Practice	Australia	Digital mental-health patients	20	Semi-structured interviews	Reflexive thematic analysis	“To qualitatively explore the video chat experiences of both participants and therapists who participated in an adaptive randomized clinical trial. The present study evaluated experiences of clinical assessments and low- and high-intensity therapist assistance delivered via video chat technology adjunctive to a transdiagnostic cognitive behaviour therapy DMH intervention program for anxiety and depression.”
Chan et al., 2020	Exploring the determinants and experiences of senior stroke patients with virtual care	Canadian Journal of Neurological Sciences	Canada	Stroke patients	23	Interviews (telephone or face-to-face)	Thematic analysis	“To explore the experiences of participants affected by stroke with home video visits (HVV) for follow-up visits to understand the determinants, barriers, and benefits associated with HVVs.”
Christensen et al., 2020	A qualitative study of patients’ and providers’ experiences with the use of videoconferences by older adults with depression	International Journal of Mental Health Nursing	Denmark	Older adults with depression	13 Patients, 12 healthcare professionals	Semi-structured interviews, focus groups	Thematic analysis	“To investigate the experiences of patients and providers regarding the use of videoconference in older patients with depression.”
Granberg et al., 2021	Medical Oncology Patient Perceptions of Telehealth Video Visits	JCO Oncology Practice	The USA	Adult oncology	12	Semi-structured interviews (telephone or face-to-face)	Qualitative content analysis	“To identify the factors influencing patient acceptability of video visits for medical oncology care before and at the onset of telehealth because of COVID-19 pandemic.”
Grinfelde, 2022	Face-to-Face with the Doctor Online: Phenomenological Analysis of Patient Experience of Teleconsultation	Human Studies	Latvia	Mixed; patients with VC experience	14	Phenomenological interview	Phenomenologically grounded qualitative research	“This study aims to determine whether teleconsultation contains the features of face-to-face encounters that are essential to clinical encounters and have been lacking in online encounters, namely the possibility of an empathetic perception of the other and the sense of embodied risk.”
Koppel et al., 2022	Exploring Nurse and Patient Experiences of Developing Rapport During Oncology Ambulatory Care Videoconferencing Visits: Qualitative Descriptive Study	Journal of Medical Internet Research	The USA	Oncology patients	10	Interviews	Content analysis	“To explore the experiences of nurses and patients participating in oncology telehealth VCVs, specifically concerning the cultivation of rapport.”
Lawson et al., 2022	Acceptability of telehealth in post-stroke memory rehabilitation: A qualitative analysis	Neuropsychological Rehabilitation	Australia	Stroke survivors	25	Semi-structured interviews	Thematic analysis	“To explore the acceptability by characterizing the experience of telerehabilitation for service providers and consumers of a memory rehabilitation program.”
Mathar et al., 2015	A qualitative study of televideo consultations for COPD patients	British Journal of Nursing	Denmark	COPD patients	6	Qualitative interviews	Systematic text condensation	“To study COPD patients’ experiences and preferences concerning hospital discharge with televideo consultations.”
Moeller et al., 2022	Patients’ experiences of home-based psychotherapy via videoconference: A qualitative study	Archives of Psychiatric Nursing	Denmark	Adult outpatients, psychotherapy	7	Semi-structured interviews	Systematic text condensation	“To explore adult outpatients’ experiences with home-based psychotherapy via videoconferencing in a Danish mental health service.”
Nissen et al., 2017	A qualitative study of COPD-patients’ experience of a telemedicine intervention	International Journal of Medical Informatics	Denmark	COPD patients	14	Semi-structured interviews (video)	Content analysis	“To investigate the patient perspective on receiving telemedicine with weekly submission of readings and regular video consultations (Net-COPD) as an alternative to visits in the respiratory outpatient clinic and investigate the role of telemedicine in the management of severe COPD.”
Nordtug et al., 2021	Patient experiences with videoconferencing as social contact and in follow-up from oncology nurses in primary health care	Health Psychology Nursing	Norway	Oncology patients	6	Semi-structured interviews (face-to-face)	Thematic analysis	“To gain knowledge of cancer patients’ lived experiences of tablet videoconferencing in municipal oncology nurses’ follow-up and contact with their family and peer network.”
Parkinson et al., 2021	‘They're getting a taste of our world': A qualitative study of people with multiple sclerosis’ experiences of accessing health care during the COVID-19 pandemic in the Australian Capital Territory	Health Expectations	Australia	Multiple sclerosis patients	8 (4 had VCs)	Semi-structured interviews (telephone or video)	Thematic analysis	“To examine the experiences of people with multiple sclerosis in accessing health care, including telehealth, during the COVID-19 pandemic in Australia following the Australian government's expansion of subsidized telehealth consultations.”
Shulver et al., 2016	'Well, if the kids can do it, I can do it': older rehabilitation patients’ experiences of telerehabilitation	Health Expectations	Australia	Older adults, rehabilitation	13	Semi-structured interviews (face-to-face)	Thematic analysis	“To study how community-dwelling older people experience rehabilitation programmes using telehealth technologies and how acceptable are telehealth technologies to older people in the context of rehabilitation.”

COPD: chronic obstructive pulmonary disease; FTF: face-to-face; VC: video consultation.

### Data synthesis

The data were analysed using thematic synthesis, as formulated by Thomas and Harden^39^; here, over the course of three stages, themes that reoccurred across the selected studies were identified. First, the extracted data were given a free line-by-line coding that summarised or described the text with a very low degree of interpretation, remaining close to the original findings.^39^ To verify the method, all three authors independently coded one study and then discussed their results. After this, each of the remaining studies was coded by the first author. Second, similar codes were discussed by the authors, grouped into descriptive themes and kept close to the original findings. Third, a more abstract and analytical discussion between the authors was held to group, explain and describe the initial descriptive themes that emerged, forming the main analytical themes.^39^

### Quality appraisal

The assessment of confidence in the findings was guided by the GRADE-CERQual (Confidence in the Evidence from Reviews of Qualitative research) approach,^40^ and performed using the iSoQ (interactive Summary of Qualitative Findings) – tool by all three authors jointly. The confidence in the evidence was based on four key components, that together contribute to an overall assessment: methodological limitations; coherence of the review findings; adequacy of the data contributing to the review findings; and relevance of the included studies to the research question of this study (Appendix 3).

## Results

**Figure 1.**
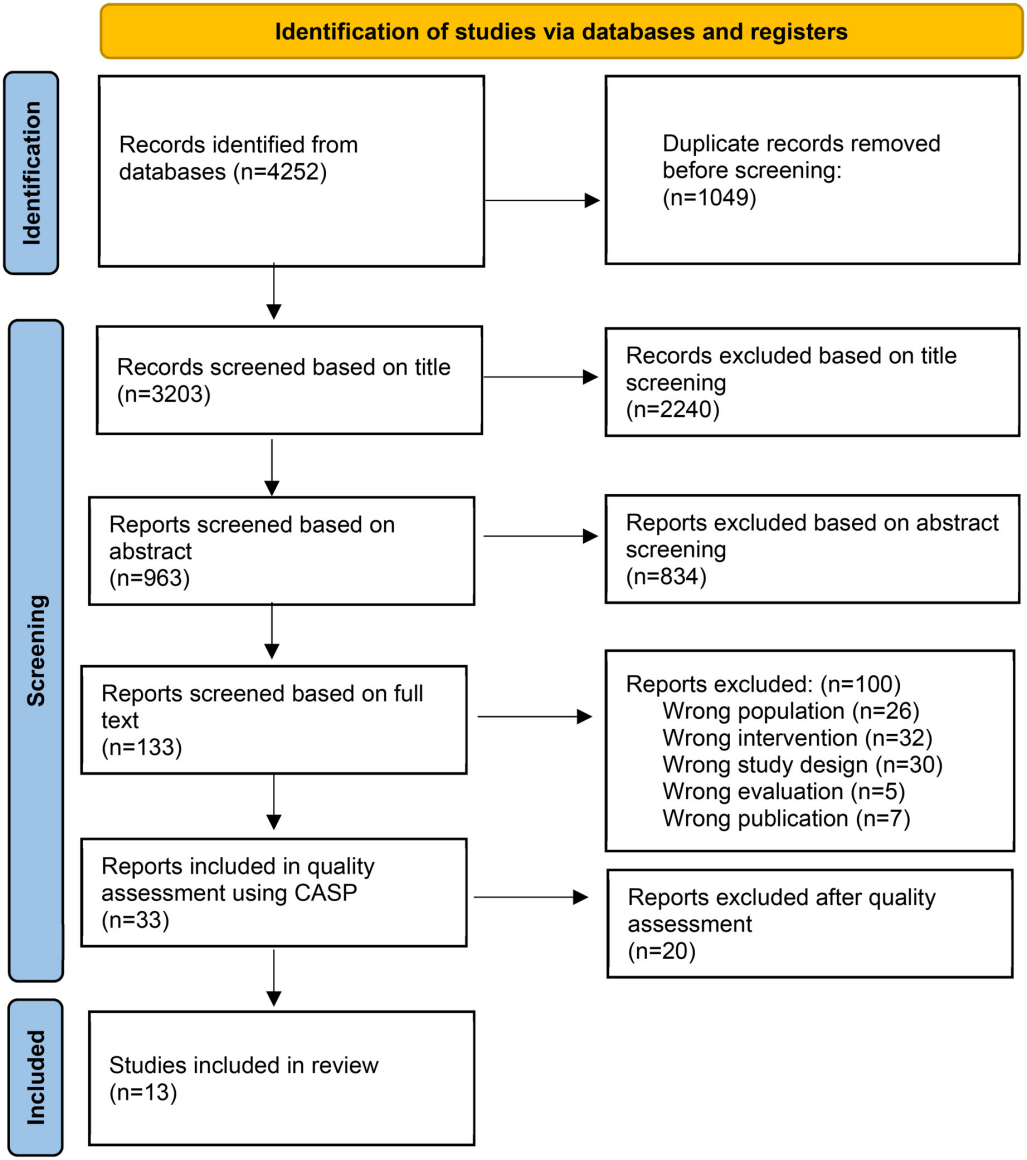
Flow chart of included and excluded studies, along with reasons for exclusion.

The search yielded 4252 publications; following the removal of duplicate citations, 3203 were studies to be screened. Following title and abstract screening, 133 were included in the full-text screening, and 33 of these were eligible for inclusion in the quality assessment. Of these, 20 studies were assessed to be poorly described, and therefore excluded; the remaining 13 studies were included in the synthesis (Figure 1).

### Demographics of included studies

The 13 included studies were conducted in six countries (Canada, the USA, Norway, Denmark, Latvia and Australia), with publication dates from 2015 to 2023. Data were collected using interviews, and thematic and content analyses were used as predominant method of analysis. The number of participants ranged from 6 to 25; they were 20 to 92 years old and the only active party in the conversation during VCs with healthcare professionals due to stroke, depression, anxiety, chronic obstructive pulmonary disease (COPD), cancer, multiple sclerosis (MS), rehabilitation or psychotherapy.

### Qualitative synthesis of findings

Thematic synthesis of adult patients’ experiences of VC with healthcare professionals identified four main themes, each with subthemes (Table 3). The synthesis did not identify any diagnostic or geographic context as dominant within the themes; however, all studies consistently supported two subthemes: ‘*Less time, effort, and travel, and more accessible*’ and ‘*Challenges to the patient-healthcare professional relationship*’.

**Table 3.** Summary of the main themes and their subthemes, with contributing references.

Suitable for less complex issues when technical problems can be solved	Feeling secure, relaxed, and having mutual focus in an equitable partnership	Limitations regarding personal needs and practical help	Increased vulnerability and lack of emotional feedback
Less time, effort, and travel, and more accessible^41–53^	A sense of privacy and security at home^41–46^^,48,51^	Does not add anything and is insufficient^42–44^^,47,48,52^	Challenges to the patient-healthcare professional relationship^41–53^
Shorter waiting times for booking appointments^41,43,46,51,53^	Having more time, relaxation, and focus^42,43,46,48,50,51,53^	More difficult to talk and ask questions^42,43,45,48^^50–53^	Patients are more vulnerable at home^43,44,46,47,50,52,53^
VCs for shorter, less complicated issues^41–44,46,47,50–53^	Feeling on the same level^42,43,45,50,53^	Physical exam and practical help are limited^41,43,45,48,49,51,52^	Less personal and provides less emotional support^41–43^^,47–49,50,52,53^
Overcoming technical problems and discomfort^41–43,46,48–51^	Positive to be able to see healthcare professionals^49–47^^,49–53^		Creates obligations and pressure^44^^49–53^
	Needs are met similarly to FTF^41–46^^,48–53^		

FTF: face-to-face; VC: video consultation.

#### Main theme 1: Suitable for less complex issues when technical problems can be solved

The first main theme relates to patients seeing VC as saving both time and energy, if used in appropriate situations and under the right circumstances, and in line with patient's capacities and condition. A patient stated:

“*Now I – to get up in the morning and make the bed and get in the shower – I’m very slow. It's at least an hour and a half before I’m ready to come down… I felt that [VCs are] a way of saving me time and effort. Physical effort*.”^41^

All studies noted that VC involves ***less time, effort and travel, and is more accessible*** – something that is worth a great deal to unwell patients.^41–53^ The reason for appreciating VC as time and effort saving sometimes differs depending on the patient's condition. One patient expressed: ‘*It´s also easier when you’ve got a baby; it's better not to have to spend an hour on transportation’,*^52^ while another patient explained: ‘*It takes a lot of energy to travel − it is awful − that noise and people around you’.*^46^ Easy access also results in a sense of control, and less anxiety and stress. There are, however, divergent experiences regarding round-the-clock access to VC; this affects the coherence of the results, since some patients believe that is possible to use VC at any time, while others do not. VC means not having to make travel arrangements, and is associated with benefits such as fewer worries and more energy for rehabilitation and healing.^41,48^ However, some feel that travel time is important for preparation before an appointment, and mention missing this during VC.^50^ Others find it easier to process information without the need to drive home after a VC.^44^ Some studies found that patients appreciate ***shorter waiting times for booking appointments*** with VC, but the circumstances that contribute to this are not described in detail, and therefore hold some uncertainty.^41,43,46,51,53^ For some, VC is preferred because it means less waiting, for example, when receiving bad or important news.^43^ Patients also feel positively about not having to sit in waiting rooms.^51,53^

Many studies noted the idea of ***VCs for shorter, less complicated issues***.^41–44^^,47,50,52^ A patient stated: ‘*Going over test results with me…he's showing me the pictures. If you were in person, you’d have to try and peek around the computer…it really is a benefit, and then like 15 minutes are over, and I feel good about myself, and I can go on about my day’.*^50^ Some patients feel that VCs are generally shorter, with less time to have a conversation, making them better suited to brief follow ups or ensuring that the patient is okay.^41,43^ As one patient described: *‘It was less personal, you know? It was a little more distant. I felt I had less time to talk to him. Less time to have a conversation’.*^41^ On the other hand, some patients believe that VCs take more time^42,43^ as one patient expressed: ‘*I felt like…there was more time…because I’ve been to doctors a lot and I just felt that the time that was spent, that I spent with the doctor was longer that if I had been in the office and she had other patients waiting*’*.*^43^ In acute situations VC is not seen as a good option – something that was highlighted by patients with COPD and depression.^42,44^ Patients with COPD mentioned the need for admission in the case of acute decline, rather than VC, although some also felt that VCs should be offered to far more unwell patients than them.^44^ The patients interviewed in that study appear to have extensive lived experience and knowledge of their disease, and seem accustomed to managing it on their own. This may have contributed to their opinion of VCs as suitable for more unwell patients, but still not for acute situations. They felt that VC does not add to their already substantial knowledge about their illness. One patient explained: ‘*You can’t call up the screen 24 hours a day. If I have a panic attack and feel poorly, all I can do is call the emergency services*’*.*^44^ Furthermore, VC is not felt to be appropriate for assessing complications or making treatment decisions, as was especially highlighted by oncology patients.^43^ For follow ups, VC is felt to be especially satisfying^42,43,46^ but not when the issues are complex.^42,43,53^ Experiences of receiving bad news relate to several of the themes in this review. Some participants feel that it is easier to hang up and not think about bad news if it is received through VC, which was felt to be inappropriate where there is a need to discuss treatment options FTF.^43,51^ As result in one of the studies, no consensus was reached on the acceptability of VC regarding the delivery of bad news.^43^

Patients described changing their attitudes to VC after trying it, and so ***overcoming technical problems and discomfort**.*^41–43^^,46,48,50,51^ This acceptance is described as accompanying familiarity with the technology.^42,43,46,51^ However, not everyone has the capacity and courage to try things like VC, which requires the ability to carefully listen to instructions, and understand and follow them.^41,42^ Some patients are dependent on other people in order to be able to use technology, and some feel that VC is only suitable for those accustomed to the internet.^41^ Technical problems lead to frustration, reduced trust during interactions, and feelings of incompetence and concern^42,43,51^ as one patient described: ‘*Before, I’d sort of stress myself out thinking, “How dumb can you be?” I’m not dumb, it's just the stroke, I just need to keep reading it*’*.*^49^ Problems mentioned relating to VC included transmission interruptions and audio-video problems,^42^ and the importance of having a reliable network was regularly highlighted.^41,46^ There is a need and expectation of technical support as backup,^41,42,48^ and some patients are concerned about their personal data and cyber security.^41,48^ However, other patients are unconcerned with technical challenges and loss of connection,^42,51^ and feel that VC is ‘real’, despite any technical problems.^49^ Patients describe feeling increased self-confidence, positive feelings and comfort when they learn to use the technology.^42,43,46,51^ This shows that many patients experience a movement during their time in contact with healthcare through VC, and one of the studies describes this movement as a new way of communicating that is essential to feeling safe and healthy when healthcare is conducted via VC.^46^ One patient expressed: ‘*I was first intimidated by the whole experience, wondering, how is this gonna work…? But by the time I…The last one I had…with my doctor, I was comfortable with it*’*.*^43^

#### Main theme 2: Feeling secure, relaxed and having mutual focus in an equitable partnership

The second main theme relates to patients feeling that VC contributes to a sense of security and equity when the conditions are right, leading to fulfilment of needs and empowered patients. Authors in one study expressed:

“*This manifest[s] in that they were able to relax and felt more empowered to express themselves better from within [the] practical comfort and easy familiarity of their own home.”*^42^

Patients feel that VC offers ***a sense of privacy and security at home**.*^41–46^^,48,51^ This is appreciated, and makes patients feel calm and empowered in the comfort of their homes, described by the authors in one study as ‘*Having the opportunity for more frequent contact means increased security*’*.*^42^ Patients describe a feeling of security associated with VC and the possibility of ‘just pressing the button’, referring to the ability to act and be in control.^44–46^ According to one patient: ‘*It means that I’m not anxious … as much as I have been before … because I can control how I am … I couldn’t before. I can get in contact with you very quickly; of course I could before, but it was in a different way…this gives a feeling of security, and when you feel secure, you don’t hyperventilate, and then you don’t get breathless so much, and that descending spiral is turned the other way round, …so you feel much better in general. And you are happier and more energetic*’*.*^45^ The feeling of security over VC is also described as an ability to distance themselves, stated by one patient as knowing that ‘*There is safety there; no one can do anything to me against my will*’*.*^51^ This feeling of security at home should, however, be interpreted with caution, because there are conditions relating to VCs that are believed to be crucial, such as support from family and the possibility of immediate contact with healthcare at any time.^43–45^ One patient described: ‘*I don’t feel like I actually have to be there for comfort or anything like that. Yeah, I guess some people might need that. For me, the comfort's more my family and that type of thing, which would be here anyway*’*.*^43^ Being at home gives a sense of privacy. Moreover, VC allows patients to avoid showing emotions in front of others, which is appreciated.^43,51^

***Having more time, relaxation and focus*** are reported as common outcomes of VC.^42,43,46,48,50,51^ Patients describe VC as being relaxed and not making them feel rushed,^43,48,53^ and appreciate the fact that it allows healthcare professionals to pay undivided attention to them by listening to and looking only at them.^42,43^ Other positives include more focused conversations,^42,43^ stated by one patient as: ‘*… you go more directly to the problem and the conversation is more concentrated because there are not things that divide your attention*’*.*^42^ Patients report ***feeling on the same level*** as a result of VC, contributing to feelings of responsibility, self-determination, and equity.^42,45,50,53^ In one of the studies this was expressed by the authors as: ‘*…they felt more equal and engaged in the co-planning of the treatment with the provider*’^42^ and a patient explained: ‘*It's just me and the doctor talking on a video visit. I’m at my office, he's in his office- and it's very personal*’*.*^43^ VC also makes patients feel that they are at the centre of their care, and able to contribute by planning alongside healthcare professionals.^42,45^ It was felt as ***positive to be able to see healthcare professionals*** through VC,^41–47^^,50–53^ and this is to be understood in contrast to phone calls with healthcare personnel. Patients report that VC improves communication as it allows patients to see attitudes, feelings and reactions,^41,42^^45–47^^,49,51–53^ along with even a sense of humour.^43^ Another aspect is hearing problems, which mean that being able to see and collaborate with a nurse via VC is felt to be positive.^46^ To be able to see each other is something that benefits the relationship between the patient and the healthcare professional.^42,47,51^

The result shows that many patients are satisfied with VC – that their ***needs are met similarly to FTF**.*^41–46^^,48–53^ The most frequent expression of this was the opinion that VC is as good as FTF,^41,42,44,52^ or that the two are not very different.^49,53^ As one patient described it: ‘*Yeah I found it fairly natural and I don’t see it as being anything different than if I was sitting in your office and we were talking across the table. So I feel it's pretty well the same thing*’*.*^49^ Some patients feel close to healthcare professionals when using VC,^53^ and report that they would choose VC again and recommend it to others.^42^ The positive feelings towards VC sometimes had a practical reason, such as the technician installing the equipment being an appreciated distraction from daily life.^44^ VC is appreciated because it is felt to be simple to use^51,53^ and a new ‘normal’.^50^

#### Main theme 3: Limitations regarding personal needs and practical help

The third main theme concerns VC restricting patients' ability to communicate effectively enough to obtain the help they need, whether that help is practical, physical or psychological, as one patient expressed it:

“*Well, it's a little bit more shorter and brief like just to make sure everything's going okay. When you’re in an office visit with the doctor, you’re more specific and asking specific questions and you’re there a little bit longer, I think, like you get more in detail.”*^43^

Some patients report that VC ***does not add anything and is insufficient**.*^42–44^^,48,52^ This is reported by patients who already know much about their illness, or who have complex problems.^42–44^ A patient stated: ‘Well*, I don’t think it would help me so much, since now I know what's expected of me, after being in and out [of hospital] four times with the same illness. So the screen wouldn’t be able to help me now*’*.*^44^ Some patients believe that a phone call can be beneficial.^47,52^ In one of the studies that investigated participants with COPD, VCs seem to be booked on a regular schedule, instead of based on the needs of patients, which may have contributed to the feeling that VCs do not add anything.^44^ Some find it ***more difficult to talk and ask questions*** during VC, and difficult to open up in front of a screen.^42,43,45,51,52^ One patient expressed: ‘*If I had something highly personal to discuss, I would feel less comfortable doing it virtually…*’*.*^50^ Difficulties when talking is described by a patient as: ‘*I also find that often in video chat I interrupt and I get interrupted more and I think it's cause either those visual cues are not the same’,*^53^ further explained by a patient as: ‘*When using video communication, you have to sit and wait for each other and this makes the conversation more formal. Meeting in person makes you feel less afraid of interrupting each other’.*^52^ In one study, the patients described FTF as easier because the appointment is longer in the office, they have more willingness to talk FTF, and it is better when questions come up.^43^ Technical problems during emotional conversations is another concern, which make patients reluctant to ask sensitive questions.^43,51^ A patient stated: ‘*Just even the possibility of maybe even bringing up an emotional concern − I might not be willing to do so for the fear that it would cut out in the middle of it and then I’m not − I’m left without support in that situation. So not having − I guess not knowing based on technology would be a disadvantage’.*^43^ Some also described not being able to conduct VCs independently as a reason for VC being insufficient,^42^ and they argued that VC should only be a supplement to FTF consultations.^48^ That ***physical exam and practical help are limited*** during VC,^41,43,48,49,51,52^ is reported to contribute to a feeling of uncertainty.^51^ Patients experience limitations concerning physical contact and exams during VC,^51^ and find it negative. The absence of resources and equipment for measuring and assessing contribute to this experience.^41,43^ The ability to fill in paperwork and obtain information and material were limited during VC, and patients generally value these things^43,52^ as explained by a patient: ‘*Let's say my diagnosis of cancer had been brought up to me on a tele video visit, I wouldn’t like that very much because I can get a lot more information in person and they can refer me to other people in the office right then. Or they can hand me a booklet or something like that they can, something right then. I mean, I know they could say, okay, I’m gonna send you a link to a PDF file or something like that. But you kind a need a little more − for something that's big like that, you kinda feel like you need a little more in-person visit*’*.*^43^ The value of a tissue being given to patients is also mentioned, and is seen as a limitation of VC.^49^ As is the impossibility of certain rehabilitation exercises.^48^ It was also felt to be difficult to consult others and access translation services during VC.^43,45^ However, the quantity of data supporting this result is not great, which could be due to the characteristics of the participants in the included studies, or the need for consultations to be more frequent within some specialties. Oncology patients find VC to be an obstacle when the need to consult others occurs during VCs; this contrasts with FTF, where the patient can be referred to or speak with other team members while in the office.^43^ The inability to use translation services during VC is also noted by oncology patients.^43^

#### Main theme 4: Increased vulnerability and lack of emotional feedback

The fourth main theme relates to patients experiencing relationships with healthcare professionals mediated solely by VC as being unsatisfactory and feeling increased personal vulnerability due to being at home with less emotional support and more responsibility. One patient described:

“*I would say that it is − in an odd way [video visits] kind of makes you a little more vulnerable to your provider because they see you in your home and sometimes in your pajamas, maybe they see − I don’t have children, but maybe they see your kids running around or they might be more attuned to parts of your personal life that you might keep confidential, whether − just in terms of making the relationship as − they probably want it to be professional or whatever. So I think that video does permit a level of vulnerability that an office visit would not.”*^43^

In 12 of the 13 included studies, the authors conclude that there exist ***challenges to the patient-healthcare professional relationship*** when using VC.^41–52^ Although VC offers patients the ability to see the health care professionals, the studies report that it is easier for them if they know each other before VC.^42,45,47,50,52^ Patients feel that it is not appropriate to meet new healthcare professionals via VC, and that knowing them beforehand is beneficial.^43,52^ One patient expressed: ‘*But I think also, having met the person in person and then relating to them is the advantage…*’*.*^48^ It is important to note that positive feelings of safety, relaxation and satisfaction relating to an appointment with a healthcare professional that is known to the patient are not always clearly dependent on VC, and could instead be an expression of the benefits of known healthcare professionals in general.^45,51,52^ Patients describe an initial FTF meeting as essential, and state that where VC is used, it must be combined with FTF, because a relationship mediated solely by VC is not good enough.^42,47^ Notably, some patients describe being able to trust an unknown physician that they have met through VC as described by the authors in one study: ‘*Some of the patients who did experience trust in the doctor when meeting her for the first time online talked about the quality of their interaction and the perceived personal investment of the doctor (for example, doctor listening attentively to their problem), which generated trust in the doctor*’.^51^ Interestingly, a patient having VC with a therapist felt more comfortable knowing that they would not meet again and expressed: ‘*yoúre telling them very personal things that you might not tell many people umm and I actually, online was okay for that because there wasn’t going to be ongoing like professional relationship*’*.*^53^ The relationship-based challenges are believed to depend on limitations in reading and understanding nonverbal signals, which are needed when building trust.^50–52^ According to some studies, the context and experience of the room in which the visit takes place can contribute to a feeling of trust and sense of a relationship with a healthcare professional during FTF visits, and this is lost with VC.^50,51,53^ In-person appointments are, according to many patients, the best way to meet with healthcare professionals and are superior to all alternatives^41–49^^,52^; when these are not possible, VC is second best.^43^

***Patients are more vulnerable at home*** is a feeling that is expressed by patients, and relates to the fact that VC allows healthcare professionals to see the interior of the patient's home, which is perceived as very personal.^43^ Some patients described feeling embarrassed to be seen before they had brushed their hair and so on.^44^ Seeing oneself on camera is described by patients as uncomfortable, and as creating a feeling of alienation.^47,52,53^ One patient described it: ‘*I do not like to watch myself on a video and prefer a telephone conversation. When using video conversation, I felt nervous and insecure. I feel uncomfortable being called up that way. So, I didn't attain what I wanted. I did not feel I could speak freely*’*.*^52^ The results show that the quality of VC depends on the ability of patients to create a quiet space at home, which is not always easy.^50,53^ Vulnerability is also seen in the importance of patients having access to a good support system, such as family at home, in the event of distressing news.^43,46^ Home is a place that, for some patients, offers security, relaxation, and calm, while for others it is related to a feeling of being insecure and vulnerable. Patients feel that VC is ***less personal and provides less emotional support***,^41–43^^,48–50,52,53^ which makes it difficult to feel compassion, comfort and reassurance from the healthcare professionals,^41,43,50,52^ and makes patients feel uncomfortable, insecure, worried and anxious.^41,52^ The idea of VC being ‘less personal’ recurs throughout the studies^41,43,48,49,52^; it is felt to sometimes leave patients with feelings of loneliness,^50^ distant^41,52^ and being unimportant,^43,52^ as one patient expressed: ‘*I feel like the tele video visits sometimes you feel like you’re next person in line, I gotta get out here. Whereas if I’m in the office, it's like okay. You feel more like you’re right there, I can ask more questions, and I don’t know. It just feels more like it's a little easier there*’*.*^43^ Missing eye contact was mentioned as one reason for this.^47,52^ In contrast, patients believe that it is easier to dismiss things and turn off if they become angry during VC compared to when meeting FTF.^52^

The results show that VC ***creates obligations and pressure***, mostly when it interferes with daily life and interrupts everyday routine.^44,52^ Some patients report feeling stuck at home in front of a screen, and that this is an unwanted obligation.^44^ A patient stated: ‘*It's good to have … but you have to be home at the agreed time … and I think I have enough on my plate at the moment … I have to go to physiotherapy and so on. If I have one of those screens … I will be stuck at home*’^44^*.* VC is also described as involving a tiresome routine and demanding long sessions, and requiring patients to be presentable early in the morning, making it difficult to stay motivated.^44,49,51^ One patient expressed: ‘*The sessions are large and intense, sometimes they’re too intense, it depends on how muddled the brain is on any particular day’.*^49^ Some patients feel pressured to master the technology required to undertake VCs and to know what to do,^49,50,53^ as well as to express themselves verbally.^49^ The authors in one study explained: ‘*The lack of physical touch also points to the increased importance of verbal communication during teleconsultation − in the absence of the physical examination, the patient's verbal account of her problem becomes very important. This puts a lot of pressure on the patient, who might not be able to give satisfactory account of her problem’.*^51^

## Discussion

This study systematically reviewed 13 qualitative studies to synthesise their findings regarding adult patients’ experiences of VC with healthcare professionals. The synthesis resulted in four main themes: (1) *suitable for less complex issues when technical problems can be solved*; (2) *feeling secure, relaxed, and having mutual focus in an equitable partnership*; (3) *limitations regarding personal needs and practical help* and (4) *increased vulnerability and lack of emotional feedback*.

The results of this study are insights from qualitative research on the varying experiences of VC, which depend on the context, personal circumstances, and individual abilities of patients. Thus, the results state that VC is sometimes experienced as being as good as FTF, but FTF is still the best option for quality healthcare. Patient experience can, presumably, vary depending on practical factors such as having home visits compared to travel long distances. If you increase the risk of attracting a deadly virus when visiting the office your motivation to use VC will likely increase. This review presents patient experiences in various specialities and over a time-period including a pandemic, with widely differing circumstances and expectations. Interpreting the results must be done with consideration that these scenarios are represented in the original studies. Results published in 2023 show that the main reason patients did not want to continue using video consultations was a preference for FTF care,^15^ regardless of disease or clinical scenario.^16^ Usability concerns were less commonly cited.^15^ When needing a prescription or receiving test results, a telephone consultations was preferred.^16^

Overall, the results show that VC contributes to feelings of security and empowerment due to its accessibility, because the home is experienced as a private, and safe place where the patient is in charge. Being at home can contribute to patients performing actions with confidence^54^ and facilitate empowered patient self-management.^55^ For some, VC can increase patients’ privacy and prevent the sense of being on display in, for example, a waiting room. These empowering feelings depend on multiple circumstances, particularly the availability of home-based support and health conditions. The need of finding a quiet place at home to create a private, safe place for the VC, is sometimes challenging, which is in line with previous research.^14,56^ Another circumstance that is recognised as crucial for patients’ feelings of security is the ability to contact healthcare whenever needed. This is not a ‘built-in’ function of VC but certainly affects patients’ sense of control. Empowered patients are more likely to feel responsible, self-determined, and equal to healthcare professionals during VC, facilitating co-planning alongside healthcare professionals and self-confidence in overcoming initial technical problems and discomfort. It seems that VC creates a sense of equality in terms of dialogue during such circumstances. Research has highlighted a shift in power dynamics from hierarchical to collaborative in the physician–patient relationship, and the complexities of vulnerabilities when using digital tools.^57^ The shifting of roles in healthcare due to the use of digital tools has for long been an area of interest emphasising the need to redesign the relational geography between actors in the healthcare process.^58^ With empowered patients, research has shown a need for healthcare professionals to transition from a role of authority to one of guidance,^57,59^ and with a shift of power and roles comes a shift of responsibility between healthcare professionals.^58,60,61^

VC contributes to patients’ feelings that they are on the same level as healthcare professionals, but there are challenges with building relationships, especially when the two have never met before. Patients find it harder to talk, ask questions and discuss complex issues during VC. When concerned with potential technical problems, patients become reluctant to discuss emotional topics. Similarly, previous research describe that even though most patients experience a sense of being in the same room as the healthcare professionals during VC ‘*the intimacy was experienced depending on the quality of the technical equipment*’*.*^62^ Unfortunately, technological difficulties relating to VC are still a challenge, even in highly developed countries. A systematic review in the Nordic countries found the technology uncertainties to be the dominant challenge present across all clinical specialisation studied,^22^ making it difficult to guarantee optimal technical conditions for VC. This reduces trust in the ‘digital relationship’ between patients and healthcare professionals, and patients argue that VCs should only supplement FTF consultations, not replace them.

Another contribution to lost trust in the patient–professional relationship when it is conducted by VC is the absence of the physical room and context that is experienced when meeting FTF. VC is experienced as less personal with eye contact missing, which gives the ability to distance oneself. This distance can be positive when it feels like a protective space − helping the person avoid pressure or persuasion *−* and allowing for better focus without external distractions. However, distance can also be a negative barrier, creating a sense of alienation between the patient and the healthcare professional.^63^ Similarly, our result show that distance can be negative when it makes things easier to dismiss and hinders a trusting relationship. When comparing differences in patient–doctor communication between FTF, and mainly VC (some telephone), a systematic review found that there is little significant difference between FTF and teleconsultation, with one exception, duration. FTF consultations were longer on average and there were fewer gaps to engage in small talk during teleconsultation.^64^ Studies show that missing small talk during VC makes the relationship more challenging.^21,65^ The experience of VC as stressed and shorter could be related to the missed small talk in our result and challenge the building of relationship with unknown healthcare professionals.

As healthcare moves from offices to homes there is a shift in power that can empower, as stated earlier, but also bring a sense of vulnerability. Patients feel more vulnerable when healthcare professionals see their home environments. Their personal space has been described by healthcare professionals as the patients holiest place^66^ that is almost sacred.^67^ In addition, the personal preparedness seems to change, resulting in embarrassment over unbrushed hair, and alienation can be caused by seeing oneself on camera. This vulnerability, combined with less emotional support from healthcare, is a risk with VC that needs to be accounted for. Research on self-view during virtual meetings indicate the need to customise the technological features individually since it is associated with the users degree of public self-consciousness,^68^ and has a negative impact on satisfaction while listening but can be neutral while speaking.^69^

Importantly, the results suggest that healthcare professionals should be sensitive, and the organisation flexible enough to succeed in supporting patients, regardless of the communication tool used. VC in healthcare is a balancing act between empowerment and vulnerability, obligation and support, distance and relationship. To deliver high-quality healthcare using VC, patient involvement and co-operation are essential.

Future research could explore patient experiences of VC, taking differences in expectations and motivations into account. The perspective of healthcare professionals is important to add in future research for a deeper understanding of the challenges in patient–professional relationship, and how to decrease the risk of vulnerability in combination with less emotional support during VC. How individual customisation of technological features affects the patients’ and healthcare professionals’ experiences of VC needs to be evaluated. Knowledge on how to best support the shift of roles among stakeholders due to VC should be further compiled and implemented in the healthcare system.

### Limitations

The limitations of this review relate to the quality of the included studies, not all of which reported on, for example, recruitment strategy and the relationships between researchers and participants. This is information that could have affected the results and has been accounted for in the assessment of confidence in the findings. Further, the included studies only report from middle- and high-income countries, the search was limited to English-language publications, and grey literature was excluded due to challenges in quality assessment and time constraints. Further, the exclusion of mixed-method studies in this review could have resulted in missed knowledge that would be important for the aim and transferability of the results. The included studies capture patient experiences of VC across a wide range of contexts, diagnoses, and healthcare professions, which enables a general conclusion across settings but limits the specificity. Transferability to age groups other than adults is limited.

## Conclusions

Patients feel that VC provides accessible care that is best suited for less complex issues, and its use is preferred when patients already know the healthcare professional they will be meeting. Taking time for small talk during VC is important to more actively improve the patient–professional relationship and increase the feeling of being seen as a person. Patients avoid asking complex and emotional questions during VC because they fear interruptions due to technical problems. Making a back-up plan in co-operation with the patient is crucial to increase the feeling of safety. In addition to overcoming technical issues, a quiet place and home-based support is important, which healthcare professionals should emphasise when suggesting VC. VCs can offer a neutral, non-hierarchical arena, and contribute to patient empowerment through a more focused dialogue. The shift of roles and responsibilities between stakeholders is necessary to support in the development of future digital healthcare. During VC, some patients experience a distance that is challenging for the relationship between the patient and the healthcare professional, but this can also strengthen the feeling of security. Patients may feel more vulnerable when revealing private spaces via video and engaging in stressful VC conversations with less support and more responsibilities compared to FTF visits – a risk healthcare stakeholders should consider.

The findings of this review indicate that the mode of communication between patients and healthcare professionals should be determined collaboratively with patients and supports system designs that enable individually tailored VC, recognising that patient vulnerability spans diverse settings and conditions. For the time being, VC should be a complement to, and not a replacement for, FTF conversations in healthcare.

## Supplemental Material

sj-docx-1-dhj-10.1177_20552076251404513 - Supplemental material for Patients’ experiences of video consultations: A qualitative systematic review Supplemental material, sj-docx-1-dhj-10.1177_20552076251404513 for Patients’ experiences of video consultations: A qualitative systematic review by Lina Ärlebrant, Robyn Schimmer and Anette Edin-Liljegren in DIGITAL HEALTH

sj-docx-2-dhj-10.1177_20552076251404513 - Supplemental material for Patients’ experiences of video consultations: A qualitative systematic review Supplemental material, sj-docx-2-dhj-10.1177_20552076251404513 for Patients’ experiences of video consultations: A qualitative systematic review by Lina Ärlebrant, Robyn Schimmer and Anette Edin-Liljegren in DIGITAL HEALTH

sj-docx-3-dhj-10.1177_20552076251404513 - Supplemental material for Patients’ experiences of video consultations: A qualitative systematic review Supplemental material, sj-docx-3-dhj-10.1177_20552076251404513 for Patients’ experiences of video consultations: A qualitative systematic review by Lina Ärlebrant, Robyn Schimmer and Anette Edin-Liljegren in DIGITAL HEALTH

sj-docx-4-dhj-10.1177_20552076251404513 - Supplemental material for Patients’ experiences of video consultations: A qualitative systematic review Supplemental material, sj-docx-4-dhj-10.1177_20552076251404513 for Patients’ experiences of video consultations: A qualitative systematic review by Lina Ärlebrant, Robyn Schimmer and Anette Edin-Liljegren in DIGITAL HEALTH

sj-docx-5-dhj-10.1177_20552076251404513 - Supplemental material for Patients’ experiences of video consultations: A qualitative systematic review Supplemental material, sj-docx-5-dhj-10.1177_20552076251404513 for Patients’ experiences of video consultations: A qualitative systematic review by Lina Ärlebrant, Robyn Schimmer and Anette Edin-Liljegren in DIGITAL HEALTH

## Appendix 1. Search strategy

**Table:**

	PUBMED	CINAHL	PSYCHINFO	WEB OF SCIENCE	COCHRANE
#1	telecommunications [mesh] OR telemedicine [mesh] OR “health personnel” [mesh] OR health [mesh] OR teleconferenc* [tiab] OR telehealth* [tiab] OR health* [tiab] OR OR telerehabilitation* [tiab] OR telecommunication* [All fields] OR “remote consultation*” [All fields] OR telemedicine [All fields] OR teleconsultation* [All fields] OR “tele consultation*” [All fields] OR tele-consultation* [All fields]	SU “health personnel” OR SU health OR teleconsultation* OR SU “tele consultation*” OR telerehabilitation* OR SU telehealth* OR TI teleconferenc* OR AB teleconferenc* OR TI health* OR AB health* OR TX telecommunication* OR TX “remote consultation*” OR TX telemedicine OR TX teleconsultation* OR TX “tele consultation*” OR TX tele-consultation*	SU “telecommunication media*” OR SU “teleconsultation*” OR SU “tele consultation*” OR SU “telemedicine” OR SU “health personnel” OR SU Health OR SU “telepsychiatry” OR SU “telepsychology” OR SU “online therapy” OR SU telerehabilitation* OR SU telehealth* OR TI “teleconferenc*” OR AB “teleconferenc*” OR TI health* OR AB health* OR TX telecommunication* OR TX “remote consultation*” OR TX telemedicine OR TX teleconsultation* OR TX “tele consultation*” OR TX tele-consultation*	ALL = (“teleconsultation*” OR “tele consultation*” OR tele-consultation* OR “health personnel” OR telemedicine OR telecommunication* OR “remote consultation*”) OR TI = (teleconferenc* OR telehealth* OR health* OR telerehabilitation*) OR AB = (teleconferenc* OR telehealth* OR health* OR telerehabilitation)	[mh telecommunications] OR [mh telemedicine] OR [mh “health personnel”] OR [mh health] OR teleconferenc*:ti,ab OR telehealth*:ti,ab OR health*:ti,ab OR telerehabilitation*:ti,ab OR telecommunication* OR “remote consultation*” OR telemedicine OR teleconsultation* OR “tele consultation*” OR tele-consultation*
­#2	videoconferencing [mesh] OR “video-based intervention*” [All fields] OR “video teleconferenc*” [All fields] OR videoconferenc* [All fields] OR “video conferenc*” [All fields] OR "video communication*"[All fields] OR "video consultation*” [All fields] OR "video telehealth*” [All fields] OR "video visit*” [All fields] OR “live video” [All fields] OR “real-time video” [All fields] OR “real time video” [All fields] OR “video call*” [All fields]	TX “video-based intervention*” OR TX “video teleconferenc*” OR TX videoconferenc* OR TX “video conferenc*” OR TX “video communication*” OR TX “video consultation*” OR TX “video telehealth*” OR TX “video visit*” OR TX “live video” OR TX “real-time video” OR TX “real time video” OR TX “video call*”	SU “audiovisual communications media” OR TX “video-based intervention*” OR TX “video teleconferenc*” OR TX videoconferenc* OR TX “video conferenc*” OR TX “video communication*” OR TX “video consultation*” OR TX “video telehealth*” OR TX “video visit*” OR TX “live video” OR TX “real-time video” OR TX “real time video” OR TX “video call*”	ALL = (videoconferenc* OR “video-based intervention*” OR “video teleconferenc*” OR videoconferenc* OR “video conferenc*” OR “video communication*” OR “video consultation*” OR “video telehealth*” OR “video visit*” OR “live video” OR “real-time video” OR “real time video” OR “video call*”	[mh videoconferencing] OR “video-based intervention*” OR video teleconferenc*” OR videoconferenc* OR “video conferenc*” OR videocommunication* OR “video consultation*” OR “video telehealth*” OR “video visit*” OR “live video” OR “real-time video” OR “real time video” OR “video call*”
#3	“health care quality, access, and evaluation” [mesh] OR “professional-patient relations” [mesh] OR “nurse-patient relations” [mesh] OR “physician-patient relations” [mesh] OR “patient satisfaction” [mesh] OR “patient preference” [mesh] OR perception [mesh] OR “patient* experience*” [All fields] OR perception* [All fields] OR "professional-patient relation*” [All fields] OR “nurse-patient relation*” [All fields] OR “physician-patient relation*” [All fields] OR “patient* satisfaction*” [All fields] OR “patient* attitude*” [All fields] OR “patient* preference*” [All fields])	MH “quality of health care+” OR TX “health care quality, access, and evaluation” OR TX “patient* experience*” OR TX perception* OR TX “professional-patient relation*” OR TX “nurse-patient relation*” OR TX “physician-patient relation*” OR TX “patient* satisfaction*” OR TX “patient* attitude*” OR TX “patient* preference*”	SU “client attitude*” OR SU “client satisfaction” OR TX “health care quality, access, and evaluation” OR TX “patient* experience*” OR TX perception* OR TX “professional-patient relation*” OR TX “nurse-patient relation*” OR TX “physician-patient relation*” OR TX “patient* satisfaction*” OR TX “patient* attitude*” OR TX “patient* preference*”	ALL = (“health care quality, access, and evaluation” OR “patient* experience*” OR perception* OR “professional-patient relation*” OR “nurse-patient relation*” OR “physician-patient relation*” OR “patient* satisfaction*” OR “patient* attitude*” OR “patient* preference*”) OR TS = (“health care” NEAR/3 (quality OR access OR evaluation))	[mh “health care quality, access, and evaluation”] OR [mh “professional-patient relations”] OR [mh “nurse-patient relations”] OR [mh “physician-patient relations”] OR [mh “patient satisfaction”] OR [mh “patient preference”] OR [mh perception] OR “patient* experience*” OR perception* OR “professional-patient relation*” OR “nurse-patient relation*” OR “physician-patient relation*” OR “patient* satisfaction*” OR “patient* attitude*” OR “patient* preference*”

## **Appendix 2.** Search strings

### 211011

**PubMed**

(telecommunications [mesh] OR telemedicine [mesh] OR “health personnel” [mesh] OR health [mesh] OR teleconferenc* [tiab] OR telehealth* [tiab] OR health* [tiab] OR telerehabilitation* [tiab] OR telecommunication* [All fields] OR “remote consultation*” [All fields] OR telemedicine [All fields] OR teleconsultation* [All fields] OR “tele consultation*” [All fields] OR tele-consultation* [All fields]) AND (videoconferencing [mesh] OR “video-based intervention*” [All fields] OR “video teleconferenc*” [All fields] OR videoconferenc* [All fields] OR “video conferenc*” [All fields] OR “video communication*"[All fields] OR “video consultation*” [All fields] OR “video telehealth*” [All fields] OR “video visit*” [All fields] OR “live video” [All fields] OR “real-time video” [All fields] OR “real time video” [All fields] OR “video call*” [All fields]) AND (“health care quality, access, and evaluation” [mesh] OR “professional-patient relations” [mesh] OR “nurse-patient relations” [mesh] OR “physician-patient relations” [mesh] OR “patient satisfaction” [mesh] OR “patient preference” [mesh] OR perception [mesh] OR “patient* experience*” [All fields] OR perception* [All fields] OR “professional-patient relation*” [All fields] OR “nurse-patient relation*” [All fields] OR “physician-patient relation*” [All fields] OR “patient* satisfaction*” [All fields] OR “patient* attitude*” [All fields] OR “patient* preference*” [All fields])

*Search 2021-10-11*

*Number of records: 1063*

**CINAHL**

(SU “health personnel” OR SU health OR teleconsultation* OR SU “tele consultation*” OR telerehabilitation* OR SU telehealth* OR TI teleconferenc* OR AB teleconferenc* OR TI health* OR AB health* OR TX telecommunication* OR TX “remote consultation*” OR TX telemedicine OR TX teleconsultation* OR TX “tele consultation*” OR TX tele-consultation*) AND (TX “video-based intervention*” OR TX “video teleconferenc*” OR TX videoconferenc* OR TX “video conferenc*” OR TX “video communication*” OR TX “video consultation*” OR TX “video telehealth*” OR TX “video visit*” OR TX “live video” OR TX “real-time video” OR TX “real time video” OR TX “video call*”) AND (MH “quality of health care+” OR TX “health care quality, access, and evaluation” OR TX “patient* experience*” OR TX perception* OR TX “professional-patient relation*” OR TX “nurse-patient relation*” OR TX “physician-patient relation*” OR TX “patient* satisfaction*” OR TX “patient* attitude*” OR TX “patient* preference*”)

*Search 2021-10-11*

*Number of records: 504*

**PsycInfo**

(SU “telecommunication media*” OR SU “teleconsultation*” OR SU “tele consultation*” OR SU “telemedicine” OR SU “health personnel” OR SU Health OR SU “telepsychiatry” OR SU “telepsychology” OR SU “online therapy” OR SU telerehabilitation* OR SU telehealth* OR TI “teleconferenc*” OR AB “teleconferenc*” OR TI health* OR AB health* OR TX telecommunication* OR TX “remote consultation*” OR TX telemedicine OR TX teleconsultation* OR TX “tele consultation*” OR TX tele-consultation*) AND (SU “audiovisual communications media” OR TX “video-based intervention*” OR TX “video teleconferenc*” OR TX videoconferenc* OR TX “video conferenc*” OR TX “video communication*” OR TX “video consultation*” OR TX “video telehealth*” OR

TX “video visit*” OR TX “live video” OR TX “real-time video” OR TX “real time video” OR TX “video call*”) AND (SU “client attitude*” OR SU “client satisfaction” OR TX “health care quality, access, and evaluation” OR TX “patient* experience*” OR TX perception* OR TX “professional-patient relation*” OR TX “nurse-patient relation*” OR TX “physician-patient relation*” OR TX “patient* satisfaction*” OR TX “patient* attitude*” OR TX “patient* preference*”)

*Search 2021-10-11*

*Number of records: 165*

**Web of Science**

(ALL = (“teleconsultation*” OR “tele consultation*” OR tele-consultation* OR “health personnel” OR telemedicine OR telecommunication* OR “remote consultation*”) OR TI = (teleconferenc* OR telehealth* OR health* OR telerehabilitation*) OR AB = (teleconferenc* OR telehealth* OR health* OR telerehabilitation)) AND (ALL = (videoconferenc* OR “video-based intervention*” OR “video teleconferenc*” OR videoconferenc* OR “video conferenc*” OR “video communication*” OR “video consultation*” OR “video telehealth*” OR “video visit*” OR “live video” OR “real-time video” OR “real time video” OR “video call*”)) AND (ALL = (“health care quality, access, and evaluation” OR “patient* experience*” OR perception* OR “professional-patient relation*” OR “nurse-patient relation*” OR “physician-patient relation*” OR “patient* satisfaction*” OR “patient* attitude*” OR “patient* preference*”) OR TS = (“health care” NEAR/3 (quality OR access OR evaluation)))

*Search 2021-10-11*

*Number of records: 497*

**Cochrane Library**

([mh telecommunications] OR [mh telemedicine] OR [mh “health personnel”] OR [mh health] OR teleconferenc*:ti,ab OR telehealth*:ti,ab OR health*:ti,ab OR telerehabilitation*:ti,ab OR telecommunication* OR “remote consultation*” OR telemedicine OR teleconsultation* OR “tele consultation*” OR tele-consultation*) AND ([mh videoconferencing] OR “video-based intervention*” OR “video teleconferenc*” OR videoconferenc* OR “video conferenc*” OR videocommunication* OR “video consultation*” OR “video telehealth*” OR “video visit*” OR “live video” OR “real-time video” OR “real time video” OR “video call*”) AND ([mh “health care quality, access, and evaluation”] OR [mh “professional-patient relations”] OR [mh “nurse-patient relations”] OR [mh “physician-patient relations”] OR [mh “patient satisfaction”] OR [mh “patient preference”] OR [mh perception] OR “patient* experience*” OR perception* OR “professional-patient relation*” OR “nurse-patient relation*” OR “physician-patient relation*” OR “patient* satisfaction*” OR “patient* attitude*” OR “patient* preference*”)

*Search 2021-10-11*

*Number of records: 391 (−1 Special Collections exkluderades) = 390*

### 240129 complementary search 2021 October – 2024 January

**PubMed**

(telecommunications [mesh] OR telemedicine [mesh] OR “health personnel” [mesh] OR health [mesh] OR teleconferenc* [tiab] OR telehealth* [tiab] OR health* [tiab] OR telerehabilitation* [tiab] OR telecommunication* [All fields] OR “remote consultation*” [All fields] OR telemedicine [All fields] OR teleconsultation* [All fields] OR “tele consultation*” [All fields] OR tele-consultation* [All fields]) AND (videoconferencing [mesh] OR “video-based intervention*” [All fields] OR “video teleconferenc*” [All fields] OR videoconferenc* [All fields] OR “video conferenc*” [All fields] OR “video communication*"[All fields] OR “video consultation*” [All fields] OR “video telehealth*” [All fields] OR “video visit*” [All fields] OR “live video” [All fields] OR “real-time video” [All fields] OR “real time video” [All fields] OR “video call*” [All fields]) AND (“health care quality, access, and evaluation” [mesh] OR “professional-patient relations” [mesh] OR “nurse-patient relations” [mesh] OR “physician-patient relations” [mesh] OR “patient satisfaction” [mesh] OR “patient preference” [mesh] OR perception [mesh] OR “patient* experience*” [All fields] OR perception* [All fields] OR “professional-patient relation*” [All fields] OR “nurse-patient relation*” [All fields] OR “physician-patient relation*” [All fields] OR “patient* satisfaction*” [All fields] OR “patient* attitude*” [All fields] OR “patient* preference*” [All fields])

*Search 2024-01-29*

*Number of records: 367 (från oktober 2021 och framåt)*

**CINAHL**

(SU “health personnel” OR SU health OR teleconsultation* OR SU “tele consultation*” OR telerehabilitation* OR SU telehealth* OR TI teleconferenc* OR AB teleconferenc* OR TI health* OR AB health* OR TX telecommunication* OR TX “remote consultation*” OR TX telemedicine OR TX teleconsultation* OR TX “tele consultation*” OR TX tele-consultation*) AND (TX “video-based intervention*” OR TX “video teleconferenc*” OR TX videoconferenc* OR TX “video conferenc*” OR TX “video communication*” OR TX “video consultation*” OR TX “video telehealth*” OR TX “video visit*” OR TX “live video” OR TX “real-time video” OR TX “real time video” OR TX “video call*”) AND (MH “quality of health care+” OR TX “health care quality, access, and evaluation” OR TX “patient* experience*” OR TX perception* OR TX “professional-patient relation*” OR TX “nurse-patient relation*” OR TX “physician-patient relation*” OR TX “patient* satisfaction*” OR TX “patient* attitude*” OR TX “patient* preference*”)

*Search: 2024-01-29*

*Number of records: 479*

**PsycInfo**

(SU “telecommunication media*” OR SU “teleconsultation*” OR SU “tele consultation*” OR SU “telemedicine” OR SU “health personnel” OR SU Health OR SU “telepsychiatry” OR SU “telepsychology” OR SU “online therapy” OR SU telerehabilitation* OR SU telehealth* OR TI “teleconferenc*” OR AB “teleconferenc*” OR TI health* OR AB health* OR TX telecommunication* OR TX “remote consultation*” OR TX telemedicine OR TX teleconsultation* OR TX “tele consultation*” OR TX tele-consultation*) AND (SU “audiovisual communications media” OR TX “video-based intervention*” OR TX “video teleconferenc*” OR TX videoconferenc* OR TX “video conferenc*” OR TX “video communication*” OR TX “video consultation*” OR TX “video telehealth*” OR

TX “video visit*” OR TX “live video” OR TX “real-time video” OR TX “real time video” OR TX “video call*”) AND (SU “client attitude*” OR SU “client satisfaction” OR TX “health care quality, access, and evaluation” OR TX “patient* experience*” OR TX perception* OR TX “professional-patient relation*” OR TX “nurse-patient relation*” OR TX “physician-patient relation*” OR TX “patient* satisfaction*” OR TX “patient* attitude*” OR TX “patient* preference*”)

*Search 20240201*

*Number of records: 94*

**Web of Science**

(ALL = (“teleconsultation*” OR “tele consultation*” OR tele-consultation* OR “health personnel” OR telemedicine OR telecommunication* OR “remote consultation*”) OR TI = (teleconferenc* OR telehealth* OR health* OR telerehabilitation*) OR AB = (teleconferenc* OR telehealth* OR health* OR telerehabilitation)) AND (ALL = (videoconferenc* OR “video-based intervention*” OR “video teleconferenc*” OR videoconferenc* OR “video conferenc*” OR “video communication*” OR “video consultation*” OR “video telehealth*” OR “video visit*” OR “live video” OR “real-time video” OR “real time video” OR “video call*”)) AND (ALL = (“health care quality, access, and evaluation” OR “patient* experience*” OR perception* OR “professional-patient relation*” OR “nurse-patient relation*” OR “physician-patient relation*” OR “patient* satisfaction*” OR “patient* attitude*” OR “patient* preference*”) OR TS = (“health care” NEAR/3 (quality OR access OR evaluation)))

*Search 2024-01-29*

*Number of records: 499 (från och med oktober 2021-2024)*

**Cochrane Library**

([mh telecommunications] OR [mh telemedicine] OR [mh “health personnel”] OR [mh health] OR teleconferenc*:ti,ab OR telehealth*:ti,ab OR health*:ti,ab OR telerehabilitation*:ti,ab OR telecommunication* OR “remote consultation*” OR telemedicine OR teleconsultation* OR “tele consultation*” OR tele-consultation*) AND ([mh videoconferencing] OR “video-based intervention*” OR “video teleconferenc*” OR videoconferenc* OR “video conferenc*” OR videocommunication* OR “video consultation*” OR “video telehealth*” OR “video visit*” OR “live video” OR “real-time video” OR “real time video” OR “video call*”) AND ([mh “health care quality, access, and evaluation”] OR [mh “professional-patient relations”] OR [mh “nurse-patient relations”] OR [mh “physician-patient relations”] OR [mh “patient satisfaction”] OR [mh “patient preference”] OR [mh perception] OR “patient* experience*” OR perception* OR “professional-patient relation*” OR “nurse-patient relation*” OR “physician-patient relation*” OR “patient* satisfaction*” OR “patient* attitude*” OR “patient* preference*”)

*Search 2024-01-29*

*Number of records: 194*

## Appendix 3. Level of confidence according to CERQual approach

**Table:**

Summarised review finding	Confidence	Methodological limitations	Coherence	Adequacy	Relevance	Studies contributing to the review finding
Main theme 1: Suitable for less complex issues when technical problems can be solved						
Theme 1. Less time, effort, and travel, and more accessible. Patients feel that VC saves time, effort, energy, and trouble. Worth a lot for the persons not feeling unwell. Easy access also led to feelings of security, control, less anxiety and stress. Not having to arrange for travels was associated with a lot of benefits	High confidence	No/very minor concerns regarding methodological limitations	No/very minor concerns regarding coherence	No/very minor concerns regarding adequacy	No/very minor concerns regarding relevance regarding relevance because the context in our review question is health care and the data supporting this theme is only from healthcare. Our population are patients, and we have only extracted data where we have been able to the identify the patient's perspective	Andrews et al., 2023; Chan et al., 2020; Christensen et al., 2020; Granberg et al., 2021; Grīnfelde, 2022; Koppel et al., 2022; Lawson et al., 2022; Mathar et al., 2015; Moeller et al., 2022; Nissen & Lindhardt, 2017; Nordtug et al., 2021; Parkinson et al., 2021; Shulver et al., 2016
Theme 2. Shorter waiting times for booking appointments. Patients feel that VC was preferred because it meant less waiting when receiving bad or important news	Moderate confidence	Moderate concerns regarding methodological limitations because only 1 ref of 3 had high reliability. 2 had unclear recruitment strategies. Nothing written on relationship	Minor concerns regarding coherence because there are only a few codes supporting this and it's not clear why some patients had a feeling of less waiting with VC	Moderate concerns regarding adequacy due to the number of studies and the data is not rich in detail regarding circumstances and context	No/very minor concerns regarding relevance because the supporting data origins from healthcare only. Data was only collected when patient perspectives were identifiable. 4 of 13 studies contribute to this theme, all from different healthcare contexts	Andrews et al., 2023; Chan et al., 2020; Granberg et al., 2021; Grīnfelde, 2022; Nordtug et al., 2021
Theme 3. VC for shorter, less complicated issues. Patient experience VC as shorter which suits brief follow ups. It was meaningful time spent. VC is not a good option in acute situations or to assess complications, treatment decisions, or sensitive subjects	High confidence	Moderate concerns regarding methodological limitations because all studies have low or moderate reliability except one (Mathar). Recruitment strategy was unknown or performed by provider, researcher and relations towards participants was not stated	Minor concerns because many experience VC as more appropriate for follow-up and short visits, less for acute situations	No/very minor concerns regarding adequacy because most data support this theme	No/very minor concerns regarding relevance because the supporting data origins from healthcare only. Data was only collected when patient perspectives were identifiable	Andrews et al., 2023; Chan et al., 2020; Christensen et al., 2020; Granberg et al., 2021; Grīnfelde, 2022; Koppel et al., 2022; Mathar et al., 2015; Moeller et al., 2022; Nordtug et al., 2021; Parkinson et al., 2021
Theme 4. Overcoming technical problems and discomfort. Patients change their attitude after trying VC. Acceptance came with familiarity but for some, help and support was a must. Technical issues led to feelings of frustration, mistrust, incompetence and concerns	High confidence	Minor concerns regarding methodological limitations because of lacking details on recruitment and researcher-participant relations. This was however assessed as having only minor implications on this theme	No/very minor concerns regarding coherence. Important to remember the individuals’ different prerequisites, some learn and some needs help	No/very minor concerns regarding adequacy because most data support this theme and are rich in details	No/very minor concerns regarding relevance because the supporting data origins from healthcare only. Data was only collected when patient perspectives were identifiable	Chan et al., 2020; Christensen et al., 2020; Granberg et al., 2021; Grīnfelde, 2022; Koppel et al., 2022; Lawson et al., 2022; Nordtug et al., 2021; Shulver et al., 2016
Main theme 2: Feeling secure, relaxed, and having mutual focus in an equitable partnership						
Theme 5. A sense of privacy and security at home. Patients feel that VC offers security and privacy in the comfort of home which is appreciated and contributes to feeling calm and empowered	High confidence	Minor concerns regarding methodological limitations because half of the studies had low or moderate reliability, and the other half high reliability	Minor concerns regarding coherence because a considerable amount data supports this theme. There is, however, also data suggesting that certain conditions must be in place for patients to feel secure with VC at home, e.g., support from family, possibility to contact at any time, the feeling of safety at home	No/very minor concerns regarding adequacy because most data support this theme	No/very minor concerns regarding relevance because the supporting data origins from healthcare only. Data was only collected when patient perspectives were identifiable	Chan et al., 2020; Christensen et al., 2020; Granberg et al., 2021; Grīnfelde, 2022; Mathar et al., 2015; Nissen & Lindhardt, 2017; Nordtug et al., 2021; Shulver et al., 2016
Theme 6. Having more time, relaxation, and focus. Patients feel relaxed in VC. Conversations are concentrated and focused	Moderate confidence	Moderate concerns regarding methodological limitations because of lacking details on recruitment and researcher-participant relations in most studies. 2 studies had high reliability	Minor concerns regarding coherence because most of the data support the theme. However, there is also data suggesting that VC meetings were experienced as shorter and with forced tempo. There is also contradictive data where patients felt relaxed and had more time available	Minor concerns regarding adequacy because of lack of supporting data. Suggests that time is context dependent	No/very minor concerns regarding relevance because the supporting data origins from healthcare only. Data was only collected when patient perspectives were identifiable	Andrews et al., 2023; Christensen et al., 2020; Granberg et al., 2021; Grīnfelde, 2022; Koppel et al., 2022; Nordtug et al., 2021; Shulver et al., 2016
Theme 7. Feeling on the same level. Patients feel self-determinate and responsible in their care while being more equal to the healthcare professionals and in co-planning	Moderate confidence	Minor concerns regarding methodological limitations because 2 of the studies have high reliability, one moderate and one low. Lacking details on recruitment and researcher–participant relations	No/very minor concerns regarding coherence	Moderate concerns regarding adequacy because few studies contribute with supporting data	No/very minor concerns regarding relevance because the supporting data origins from healthcare only. Data was only collected when patient perspectives were identifiable. 4 studies contributed to this theme, all from different healthcare contexts	Andrews et al., 2023; Christensen et al., 2020; Granberg et al., 2021; Koppel et al., 2022; Nissen & Lindhardt, 2017
Theme 8. Positive to be able to see healthcare professionals. Patients appreciate to see the healthcare professionals. It improves the communication and led to control and relational benefits	High confidence	Minor concerns regarding methodological limitations because some of the studies have low reliability and lack details on recruitment and researcher-participant relations. However, 10 of 13 studies contributed data for this theme	No/very minor concerns regarding coherence. Remember that some found it negative to see themselves on camera	No/very minor concerns regarding adequacy because most of the data supported this theme	No/very minor concerns regarding relevance because the supporting data origins from healthcare only. Data was only collected when patient perspectives were identifiable	Andrews et al., 2023; Chan et al., 2020; Christensen et al., 2020; Granberg et al., 2021; Grīnfelde, 2022; Koppel et al., 2022; Lawson et al., 2022; Mathar et al., 2015; Moeller et al., 2022; Nissen & Lindhardt, 2017; Nordtug et al., 2021; Parkinson et al., 2021
Theme 9. Needs are met similarly to FTF. Patients is satisfied with VC, would choose it again and recommend it, even to examine visual symptoms.VC is found personal and no different than FTF	Moderate confidence	Moderate concerns regarding methodological limitations because most of the studies were assessed as having moderate reliability	Minor concerns regarding coherence because a lot of data supports the theme, however, there are also contradictory data where patients expressed experiences pointing in opposite directions	No/very minor concerns regarding adequacy	No/very minor concerns regarding relevance because the supporting data origins from healthcare only. Data was only collected when patient perspectives were identifiable	Andrews et al., 2023; Chan et al., 2020; Christensen et al., 2020; Granberg et al., 2021; Grīnfelde, 2022; Koppel et al., 2022; Lawson et al., 2022; Mathar et al., 2015; Moeller et al., 2022; Nissen & Lindhardt, 2017; Nordtug et al., 2021; Shulver et al., 2016
Main theme 3: Limitations regarding personal needs and practical help						
Theme 10. Does not add anything and is insufficient. VC is not enough and a disadvantage for complex needs	High confidence	Minor concerns regarding methodological limitations because the studies have high or moderate reliability but are only 5	No/very minor concerns regarding coherence because this seem to be true for patients with a lot of knowledge about their own disease and when VC is booked ‘just because’ without a need for it	Minor concerns regarding adequacy because of the quantity of the data	No/very minor concerns regarding relevance because the supporting data origins from healthcare only. Data was only collected when patient perspectives were identifiable	Christensen et al., 2020; Granberg et al., 2021; Mathar et al., 2015; Moeller et al., 2022; Parkinson et al., 2021; Shulver et al., 2016
Theme 11. More difficult to talk and ask questions. It is difficult to go into complex issues, ask questions, and open up. Disturbing technical problems prevent emotional conversations. Not being able to call whenever needed is a drawback	High confidence	Minor concerns regarding methodological limitations because of the contributing studies to this theme, 3 studies have moderate reliability and 2 have high reliability	No/very minor concerns regarding coherence because this is true for some patient while others experience it as easier to talk over VC. There is a theme saying that VC is only suitable for shorter, less complicated issues which also strengthen this theme	Minor concerns regarding adequacy because although only a few studies support the theme, this is likely to be an experience that some patients have	No/very minor concerns regarding relevance	Andrews et al., 2023; Christensen et al., 2020; Granberg et al., 2021; Grīnfelde, 2022; Koppel et al., 2022; Moeller et al., 2022; Nissen & Lindhardt, 2017; Shulver et al., 2016
Theme 12. Physical exam and practical help are limited. It is negative that physical exams and practical help with paperwork and information material is limited since it is important. To consult others and get translation service is not possible in VC	High confidence	Minor concerns regarding methodological limitations because of variations in quality. Half of the studies had high reliability, one moderate and one low. Lacking details on recruitment and researcher-participant relations	No/very minor concerns regarding coherence	Minor concerns regarding adequacy because of the large quantity of data. Concerns that this theme might be pruned to be diagnose specific	No/very minor concerns regarding relevance because the supporting data origins from healthcare only. Data was only collected when patient perspectives were identifiable	Chan et al., 2020; Granberg et al., 2021; Grīnfelde, 2022; Lawson et al., 2022; Moeller et al., 2022; Nissen & Lindhardt, 2017; Shulver et al., 2016
Main theme 4: Increased vulnerability and lack of emotional feedback						
Theme 13. Challenges to the patient-healthcare professional relationship. It is beneficial to have an established relationship before VC and to meet first FTF. A relationship only on VC is not enough and patients prefer FTF consultations over VC	High confidence	Minor concerns regarding methodological limitations because only half of the studies have high reliability	No/very minor concerns regarding coherence because there is substantial data supporting the patient experience expressed in this theme	No/very minor concerns regarding adequacy	No/very minor concerns regarding relevance because the supporting data origins from healthcare only. Data was only collected when patient perspectives were identifiable	Andrews et al., 2023; Chan et al., 2020; Christensen et al., 2020; Granberg et al., 2021; Grīnfelde, 2022; Koppel et al., 2022; Lawson et al., 2022; Mathar et al., 2015; Moeller et al., 2022; Nissen & Lindhardt, 2017; Nordtug et al., 2021; Parkinson et al., 2021; Shulver et al., 2016
Theme 14. Patients are more vulnerable at home. A good support system, like family, is crucial and patients feel more vulnerable at home. Home is a private space, and some did not like to see themselves on camera	High confidence	Moderate concerns regarding methodological limitations because of variations in quality. Two studies were assessed as having low reliability, two with high reliability and the rest with moderate reliability	No/very minor concerns regarding coherence because the patients are more vulnerable at home, because it's revealing more about their personal life. But on the other hand, the security of being at home can also be experienced. This is two-folded	Minor concerns regarding adequacy because there is substantial data supporting this theme	No/very minor concerns regarding relevance because the supporting data origins from healthcare only. Data was only collected when patient perspectives were identifiable	Andrews et al., 2023; Granberg et al., 2021; Koppel et al., 2022; Mathar et al., 2015; Moeller et al., 2022; Nordtug et al., 2021; Parkinson et al., 2021
Theme 15. Less personal and provides less emotional support. VC is less personal, and some feel unimportant, rushed, just like the next person in line. To interpret reactions is difficult and to feel compassion, comfort, and reassurance. Patients feel insecure, distant, worried and anxious during VC	High confidence	Minor concerns regarding methodological limitations because of lacking details on recruitment and researcher-participant relations. Two studies with high reliability. Missing information on recruitment strategies was assessed as having low impact on this particular theme	No/very minor concerns regarding coherence	No/very minor concerns regarding adequacy because there is substantial data supporting this theme	No/very minor concerns regarding relevance because the supporting data origins from healthcare only. Data was only collected when patient perspectives were identifiable	Andrews et al., 2023; Chan et al., 2020; Christensen et al., 2020; Granberg et al., 2021; Koppel et al., 2022; Lawson et al., 2022; Moeller et al., 2022; Parkinson et al., 2021; Shulver et al., 2016
Theme 16. Creates obligations and pressure. VC interfere with daily life which is disturbing and to feel stuck in front of the screen at home is an unwanted obligation and a tiresome customary routine	Moderate confidence	Minor concerns regarding methodological limitations because of variations in quality. 3 of the studies have high reliability	Minor concerns regarding coherence. Data shows the importance of VC supporting a specific purpose and it should not to be implemented without a reason or need. There is also contradictive data supporting the opposite, patients experiencing VC as relaxing	Minor concerns regarding adequacy because only a few studies support this data	No/very minor concerns regarding relevance	Andrews et al., 2023; Grīnfelde, 2022; Koppel et al., 2022; Mathar et al., 2015; Moeller et al., 2022; Shulver et al., 2016
