# Supplementary material for: Patients’ experiences of video consultations: A qualitative systematic review
Source: Digit Health. 2026 Jan 5;12:20552076251404513. doi: 10.1177/20552076251404513 (PMC12775311; doi:10.1177/20552076251404513)
Supplement: sj-docx-1-dhj-10.1177_20552076251404513 - Supplemental material for Patients’ experiences of video consultations: A qualitative systematic review [file sj-docx-1-dhj-10.1177_20552076251404513.docx]

# **Appendix 1.** Search strategy

|  | PUBMED | CINAHL | PSYCHINFO | WEB OF SCIENCE | COCHRANE |
| --- | --- | --- | --- | --- | --- |
| #1 | telecommunications [mesh] OR telemedicine [mesh] OR “health personnel” [mesh] OR health [mesh] OR  teleconferenc* [tiab] OR telehealth* [tiab] OR health* [tiab] OR  OR telerehabilitation* [tiab] OR  telecommunication* [All fields] OR “remote consultation*” [All fields] OR telemedicine [All fields] OR teleconsultation* [All fields] OR “tele consultation*” [All fields] OR tele-consultation* [All fields] | SU “health personnel” OR SU health OR teleconsultation* OR SU “tele consultation*” OR telerehabilitation* OR SU telehealth* OR  TI teleconferenc* OR AB teleconferenc* OR TI health* OR AB health* OR  TX telecommunication* OR  TX “remote consultation*” OR TX telemedicine OR TX teleconsultation* OR TX “tele consultation*” OR TX tele-consultation* | SU “telecommunication media*” OR  SU “teleconsultation*” OR SU “tele consultation*” OR SU “telemedicine” OR SU “health personnel” OR SU Health OR SU "telepsychiatry" OR SU "telepsychology" OR SU “online therapy” OR SU telerehabilitation* OR SU telehealth* OR  TI “teleconferenc*” OR AB “teleconferenc*” OR TI health* OR AB health* OR  TX telecommunication* OR TX “remote consultation*” OR TX telemedicine OR TX teleconsultation* OR TX “tele consultation*” OR TX tele-consultation* | ALL=(“teleconsultation*” OR “tele consultation*” OR tele-consultation* OR “health personnel” OR telemedicine OR telecommunication* OR “remote consultation*”) OR  TI=(teleconferenc* OR telehealth* OR health* OR telerehabilitation*)  OR  AB=( teleconferenc* OR telehealth* OR health* OR telerehabilitation) | [mh telecommunications] OR [mh telemedicine] OR [mh “health personnel”] OR [mh health] OR  teleconferenc*:ti,ab OR telehealth*:ti,ab OR health*:ti,ab OR telerehabilitation*:ti,ab OR  telecommunication* OR “remote consultation*” OR telemedicine OR teleconsultation* OR “tele consultation*” OR tele-consultation* |
| ­#2 | videoconferencing [mesh] OR  ”video-based intervention*” [All fields] OR  “video teleconferenc*” [All fields] OR  videoconferenc* [All fields] OR “video conferenc*” [All fields] OR  "video communication*"[All fields] OR  "video consultation*" [All fields] OR  "video telehealth*" [All fields] OR  "video visit*" [All fields] OR “live video” [All fields] OR “real-time video” [All fields] OR “real time video” [All fields] OR “video call*” [All fields] | TX ”video-based intervention*” OR  TX “video teleconferenc*” OR  TX videoconferenc* OR TX “video conferenc*” OR  TX "video communication*" OR  TX "video consultation*" OR  TX "video telehealth*" OR  TX "video visit*” OR TX “live video” OR TX “real-time video” OR TX “real time video” OR TX “video call*” | SU ”audiovisual communications media” OR  TX ”video-based intervention*” OR TX “video teleconferenc*” OR TX videoconferenc* OR TX “video conferenc*” OR TX "video communication*" OR  TX "video consultation*" OR  TX "video telehealth*" OR  TX "video visit*" OR TX “live video” OR TX “real-time video” OR TX “real time video” OR TX “video call*” | ALL=(videoconferenc* OR “video-based intervention*” OR “video teleconferenc*” OR videoconferenc* OR “video conferenc*” OR "video communication*" OR "video consultation*" OR "video telehealth*" OR "video visit*" OR “live video” OR “real-time video” OR “real time video” OR “video call*” | [mh videoconferencing] OR  “video-based intervention*” OR video teleconferenc*” OR videoconferenc* OR “video conferenc*” OR videocommunication* OR “video consultation*” OR “video telehealth*” OR “video visit*” OR “live video” OR “real-time video” OR “real time video” OR “video call*” |
| #3 | “health care quality, access, and evaluation” [mesh] OR "professional-patient relations" [mesh] OR “nurse-patient relations” [mesh] OR “physician-patient relations” [mesh] OR “patient satisfaction" [mesh] OR “patient preference” [mesh] OR  perception [mesh]  OR “patient* experience*” [All fields] OR perception* [All fields] OR  "professional-patient relation*" [All fields] OR “nurse-patient relation*” [All fields] OR “physician-patient relation*” [All fields] OR “patient* satisfaction*” [All fields] OR “patient* attitude*” [All fields] OR “patient* preference*” [All fields]) | MH “quality of health care+” OR TX “health care quality, access, and evaluation” OR TX “patient* experience*" OR TX perception* OR TX "professional-patient relation*" OR TX “nurse-patient relation*” OR TX “physician-patient relation*” OR TX “patient* satisfaction*” OR TX “patient* attitude*” OR TX “patient* preference*” | SU “client attitude*” OR SU “client satisfaction” OR TX “health care quality, access, and evaluation” OR TX “patient* experience*" OR TX perception* OR TX "professional-patient relation*" OR TX “nurse-patient relation*” OR TX “physician-patient relation*” OR TX “patient* satisfaction*” OR TX “patient* attitude*” OR TX “patient* preference*” | ALL=(“health care quality, access, and evaluation” OR “patient* experience*” OR perception* OR "professional-patient relation*" OR “nurse-patient relation*” OR “physician-patient relation*” OR “patient* satisfaction*" OR “patient* attitude*” OR “patient* preference*”) OR TS=(“health care” NEAR/3 (quality OR access OR evaluation)) | [mh “health care quality, access, and evaluation”] OR [mh “professional-patient relations”] OR [mh “nurse-patient relations”] OR [mh “physician-patient relations”] OR [mh “patient satisfaction”] OR [mh “patient preference”] OR [mh perception] OR  “patient* experience*” OR perception* OR “professional-patient relation*” OR “nurse-patient relation*” OR “physician-patient relation*” OR “patient* satisfaction*” OR “patient* attitude*” OR “patient* preference*” |
