# Supplementary material for: Patients’ experiences of video consultations: A qualitative systematic review
Source: Digit Health. 2026 Jan 5;12:20552076251404513. doi: 10.1177/20552076251404513 (PMC12775311; doi:10.1177/20552076251404513)

# **Appendix 2.** Search strings

# 211011

**PubMed**

(telecommunications [mesh] OR telemedicine [mesh] OR “health personnel” [mesh] OR health [mesh] OR teleconferenc* [tiab] OR telehealth* [tiab] OR health* [tiab] OR telerehabilitation* [tiab] OR telecommunication* [All fields] OR “remote consultation*” [All fields] OR telemedicine [All fields] OR teleconsultation* [All fields] OR “tele consultation*” [All fields] OR tele-consultation* [All fields]) AND (videoconferencing [mesh] OR ”video-based intervention*” [All fields] OR “video teleconferenc*” [All fields] OR videoconferenc* [All fields] OR “video conferenc*” [All fields] OR "video communication*"[All fields] OR "video consultation*" [All fields] OR "video telehealth*" [All fields] OR "video visit*" [All fields] OR “live video” [All fields] OR “real-time video” [All fields] OR “real time video” [All fields] OR “video call*” [All fields]) AND (“health care quality, access, and evaluation” [mesh] OR "professional-patient relations" [mesh] OR “nurse-patient relations” [mesh] OR “physician-patient relations” [mesh] OR “patient satisfaction" [mesh] OR “patient preference” [mesh] OR perception [mesh] OR “patient* experience*” [All fields] OR perception* [All fields] OR "professional-patient relation*" [All fields] OR “nurse-patient relation*” [All fields] OR “physician-patient relation*” [All fields] OR “patient* satisfaction*” [All fields] OR “patient* attitude*” [All fields] OR “patient* preference*” [All fields])

*Search 2021-10-11*

*Number of records: 1063*


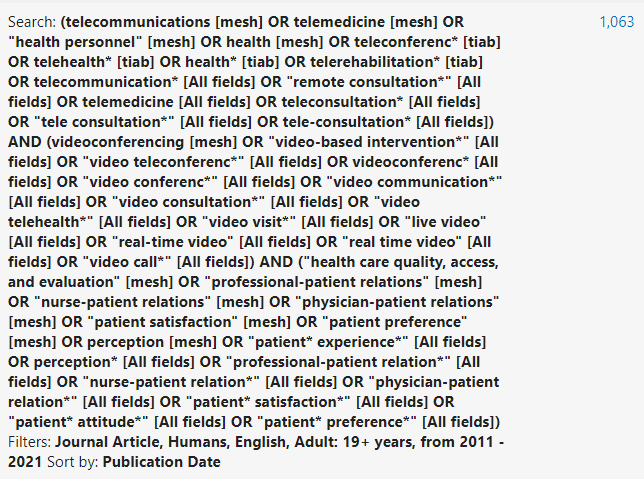


**Cinahl**

(SU “health personnel” OR SU health OR teleconsultation* OR SU “tele consultation*” OR telerehabilitation* OR SU telehealth* OR TI teleconferenc* OR AB teleconferenc* OR TI health* OR AB health* OR TX telecommunication* OR TX “remote consultation*” OR TX telemedicine OR TX teleconsultation* OR TX “tele consultation*” OR TX tele-consultation*) AND (TX ”video-based intervention*” OR TX “video teleconferenc*” OR TX videoconferenc* OR TX “video conferenc*” OR TX "video communication*" OR TX "video consultation*" OR TX "video telehealth*" OR TX "video visit*” OR TX “live video” OR TX “real-time video” OR TX “real time video” OR TX “video call*”) AND (MH “quality of health care+” OR TX “health care quality, access, and evaluation” OR TX “patient* experience*" OR TX perception* OR TX "professional-patient relation*" OR TX “nurse-patient relation*” OR TX “physician-patient relation*” OR TX “patient* satisfaction*” OR TX “patient* attitude*” OR TX “patient* preference*”)

*Search 2021-10-11*

*Number of records: 504*


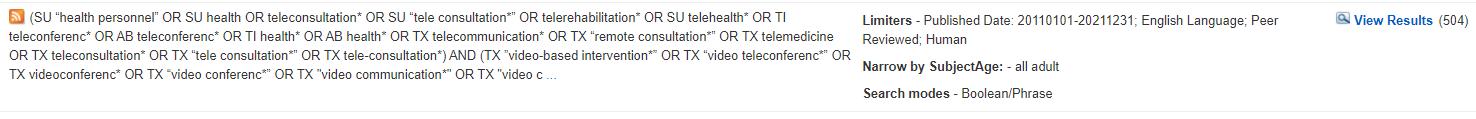


**Psycinfo**

(SU “telecommunication media*” OR SU “teleconsultation*” OR SU “tele consultation*” OR SU “telemedicine” OR SU “health personnel” OR SU Health OR SU "telepsychiatry" OR SU "telepsychology" OR SU “online therapy” OR SU telerehabilitation* OR SU telehealth* OR TI “teleconferenc*” OR AB “teleconferenc*” OR TI health* OR AB health* OR TX telecommunication* OR TX “remote consultation*” OR TX telemedicine OR TX teleconsultation* OR TX “tele consultation*” OR TX tele-consultation*) AND (SU ”audiovisual communications media” OR TX ”video-based intervention*” OR TX “video teleconferenc*” OR TX videoconferenc* OR TX “video conferenc*” OR TX "video communication*" OR TX "video consultation*" OR TX "video telehealth*" OR

TX "video visit*" OR TX “live video” OR TX “real-time video” OR TX “real time video” OR TX “video call*”) AND (SU “client attitude*” OR SU “client satisfaction” OR TX “health care quality, access, and evaluation” OR TX “patient* experience*" OR TX perception* OR TX "professional-patient relation*" OR TX “nurse-patient relation*” OR TX “physician-patient relation*” OR TX “patient* satisfaction*” OR TX “patient* attitude*” OR TX “patient* preference*”)

*Search 2021-10-11*

*Number of records: 165*


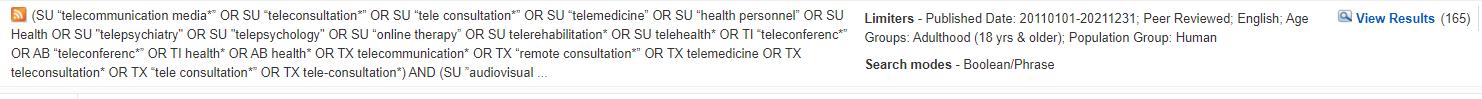


**Web of science**

(ALL=(“teleconsultation*” OR “tele consultation*” OR tele-consultation* OR “health personnel” OR telemedicine OR telecommunication* OR “remote consultation*”) OR TI=(teleconferenc* OR telehealth* OR health* OR telerehabilitation*) OR AB=( teleconferenc* OR telehealth* OR health* OR telerehabilitation)) AND (ALL=(videoconferenc* OR “video-based intervention*” OR “video teleconferenc*” OR videoconferenc* OR “video conferenc*” OR "video communication*" OR "video consultation*" OR "video telehealth*" OR "video visit*" OR “live video” OR “real-time video” OR “real time video” OR “video call*”)) AND (ALL=(“health care quality, access, and evaluation” OR “patient* experience*” OR perception* OR "professional-patient relation*" OR “nurse-patient relation*” OR “physician-patient relation*” OR “patient* satisfaction*" OR “patient* attitude*” OR “patient* preference*”) OR TS=(“health care” NEAR/3 (quality OR access OR evaluation)))

*Search 2021-10-11*

*Number of records: 497*


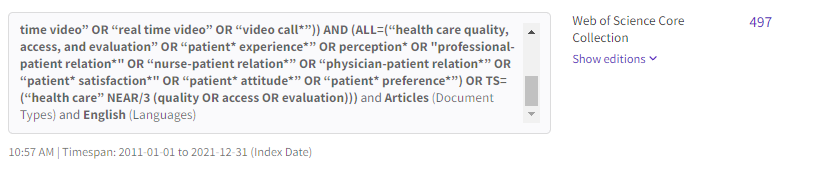


**Cochrane Library**

([mh telecommunications] OR [mh telemedicine] OR [mh “health personnel”] OR [mh health] OR teleconferenc*:ti,ab OR telehealth*:ti,ab OR health*:ti,ab OR telerehabilitation*:ti,ab OR telecommunication* OR “remote consultation*” OR telemedicine OR teleconsultation* OR “tele consultation*” OR tele-consultation*) AND ([mh videoconferencing] OR “video-based intervention*” OR “video teleconferenc*” OR videoconferenc* OR “video conferenc*” OR videocommunication* OR “video consultation*” OR “video telehealth*” OR “video visit*” OR “live video” OR “real-time video” OR “real time video” OR “video call*”) AND ([mh “health care quality, access, and evaluation”] OR [mh “professional-patient relations”] OR [mh “nurse-patient relations”] OR [mh “physician-patient relations”] OR [mh “patient satisfaction”] OR [mh “patient preference”] OR [mh perception] OR “patient* experience*” OR perception* OR “professional-patient relation*” OR “nurse-patient relation*” OR “physician-patient relation*” OR “patient* satisfaction*” OR “patient* attitude*” OR “patient* preference*”)

Search 2021-10-11

Number of records: 391 (-1 Special Collections exkluderades) = 390


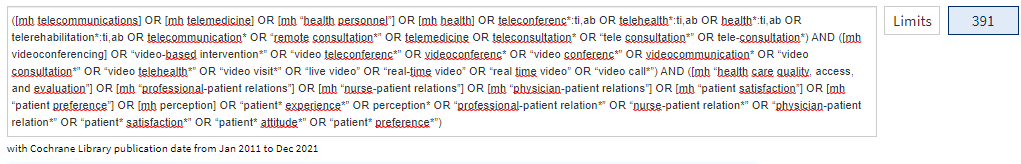


# 240129 Complementary search 2021 October – 2024 January

**Pubmed**

(telecommunications [mesh] OR telemedicine [mesh] OR “health personnel” [mesh] OR health [mesh] OR teleconferenc* [tiab] OR telehealth* [tiab] OR health* [tiab] OR telerehabilitation* [tiab] OR telecommunication* [All fields] OR “remote consultation*” [All fields] OR telemedicine [All fields] OR teleconsultation* [All fields] OR “tele consultation*” [All fields] OR tele-consultation* [All fields]) AND (videoconferencing [mesh] OR ”video-based intervention*” [All fields] OR “video teleconferenc*” [All fields] OR videoconferenc* [All fields] OR “video conferenc*” [All fields] OR "video communication*"[All fields] OR "video consultation*" [All fields] OR "video telehealth*" [All fields] OR "video visit*" [All fields] OR “live video” [All fields] OR “real-time video” [All fields] OR “real time video” [All fields] OR “video call*” [All fields]) AND (“health care quality, access, and evaluation” [mesh] OR "professional-patient relations" [mesh] OR “nurse-patient relations” [mesh] OR “physician-patient relations” [mesh] OR “patient satisfaction" [mesh] OR “patient preference” [mesh] OR perception [mesh] OR “patient* experience*” [All fields] OR perception* [All fields] OR "professional-patient relation*" [All fields] OR “nurse-patient relation*” [All fields] OR “physician-patient relation*” [All fields] OR “patient* satisfaction*” [All fields] OR “patient* attitude*” [All fields] OR “patient* preference*” [All fields])

Search 2024-01-29

Number of records: 367 (från oktober 2021 och framåt)


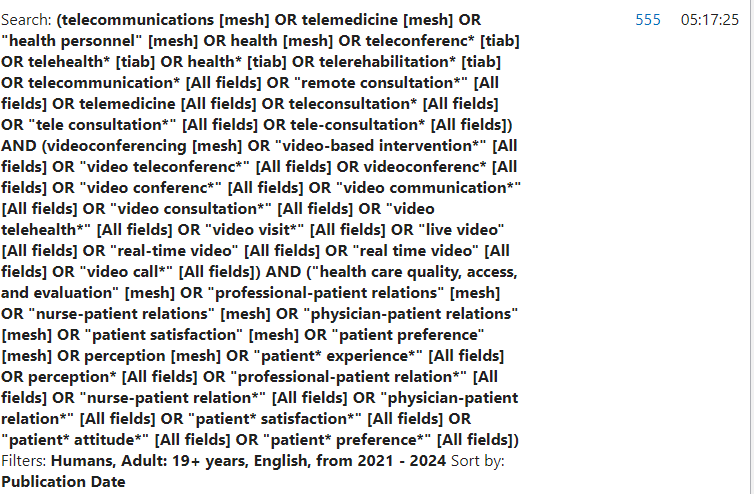


**Cinahl**

(SU “health personnel” OR SU health OR teleconsultation* OR SU “tele consultation*” OR telerehabilitation* OR SU telehealth* OR TI teleconferenc* OR AB teleconferenc* OR TI health* OR AB health* OR TX telecommunication* OR TX “remote consultation*” OR TX telemedicine OR TX teleconsultation* OR TX “tele consultation*” OR TX tele-consultation*) AND (TX ”video-based intervention*” OR TX “video teleconferenc*” OR TX videoconferenc* OR TX “video conferenc*” OR TX "video communication*" OR TX "video consultation*" OR TX "video telehealth*" OR TX "video visit*” OR TX “live video” OR TX “real-time video” OR TX “real time video” OR TX “video call*”) AND (MH “quality of health care+” OR TX “health care quality, access, and evaluation” OR TX “patient* experience*" OR TX perception* OR TX "professional-patient relation*" OR TX “nurse-patient relation*” OR TX “physician-patient relation*” OR TX “patient* satisfaction*” OR TX “patient* attitude*” OR TX “patient* preference*”)

Search: 2024-01-29

Number of records: 479


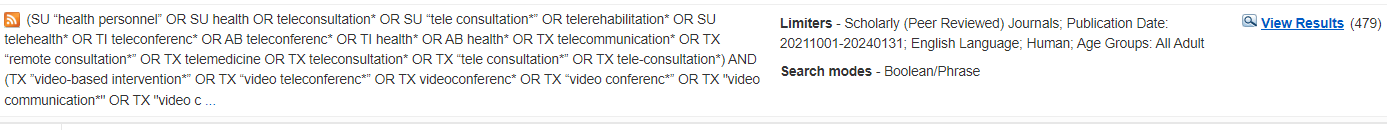


**Psycinfo**

(SU “telecommunication media*” OR SU “teleconsultation*” OR SU “tele consultation*” OR SU “telemedicine” OR SU “health personnel” OR SU Health OR SU "telepsychiatry" OR SU "telepsychology" OR SU “online therapy” OR SU telerehabilitation* OR SU telehealth* OR TI “teleconferenc*” OR AB “teleconferenc*” OR TI health* OR AB health* OR TX telecommunication* OR TX “remote consultation*” OR TX telemedicine OR TX teleconsultation* OR TX “tele consultation*” OR TX tele-consultation*) AND (SU ”audiovisual communications media” OR TX ”video-based intervention*” OR TX “video teleconferenc*” OR TX videoconferenc* OR TX “video conferenc*” OR TX "video communication*" OR TX "video consultation*" OR TX "video telehealth*" OR

TX "video visit*" OR TX “live video” OR TX “real-time video” OR TX “real time video” OR TX “video call*”) AND (SU “client attitude*” OR SU “client satisfaction” OR TX “health care quality, access, and evaluation” OR TX “patient* experience*" OR TX perception* OR TX "professional-patient relation*" OR TX “nurse-patient relation*” OR TX “physician-patient relation*” OR TX “patient* satisfaction*” OR TX “patient* attitude*” OR TX “patient* preference*”)

Search 20240201

Number of records: 94

**
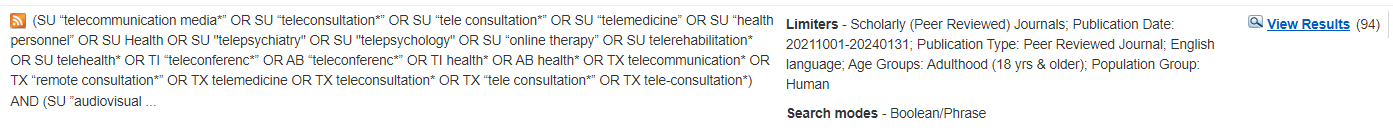
**

**Web of science**

(ALL=(“teleconsultation*” OR “tele consultation*” OR tele-consultation* OR “health personnel” OR telemedicine OR telecommunication* OR “remote consultation*”) OR TI=(teleconferenc* OR telehealth* OR health* OR telerehabilitation*) OR AB=( teleconferenc* OR telehealth* OR health* OR telerehabilitation)) AND (ALL=(videoconferenc* OR “video-based intervention*” OR “video teleconferenc*” OR videoconferenc* OR “video conferenc*” OR "video communication*" OR "video consultation*" OR "video telehealth*" OR "video visit*" OR “live video” OR “real-time video” OR “real time video” OR “video call*”)) AND (ALL=(“health care quality, access, and evaluation” OR “patient* experience*” OR perception* OR "professional-patient relation*" OR “nurse-patient relation*” OR “physician-patient relation*” OR “patient* satisfaction*" OR “patient* attitude*” OR “patient* preference*”) OR TS=(“health care” NEAR/3 (quality OR access OR evaluation)))

Search 2024-01-29

Number of records: 499 (från och med oktober 2021-2024)


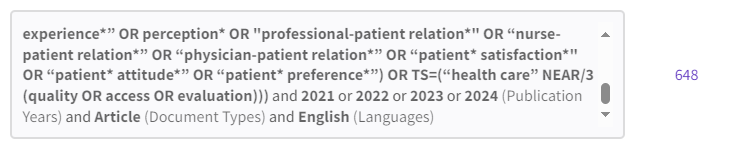


**Cochrane Library**

([mh telecommunications] OR [mh telemedicine] OR [mh “health personnel”] OR [mh health] OR teleconferenc*:ti,ab OR telehealth*:ti,ab OR health*:ti,ab OR telerehabilitation*:ti,ab OR telecommunication* OR “remote consultation*” OR telemedicine OR teleconsultation* OR “tele consultation*” OR tele-consultation*) AND ([mh videoconferencing] OR “video-based intervention*” OR “video teleconferenc*” OR videoconferenc* OR “video conferenc*” OR videocommunication* OR “video consultation*” OR “video telehealth*” OR “video visit*” OR “live video” OR “real-time video” OR “real time video” OR “video call*”) AND ([mh “health care quality, access, and evaluation”] OR [mh “professional-patient relations”] OR [mh “nurse-patient relations”] OR [mh “physician-patient relations”] OR [mh “patient satisfaction”] OR [mh “patient preference”] OR [mh perception] OR “patient* experience*” OR perception* OR “professional-patient relation*” OR “nurse-patient relation*” OR “physician-patient relation*” OR “patient* satisfaction*” OR “patient* attitude*” OR “patient* preference*”)

Search 2024-01-29

Number of records: 194


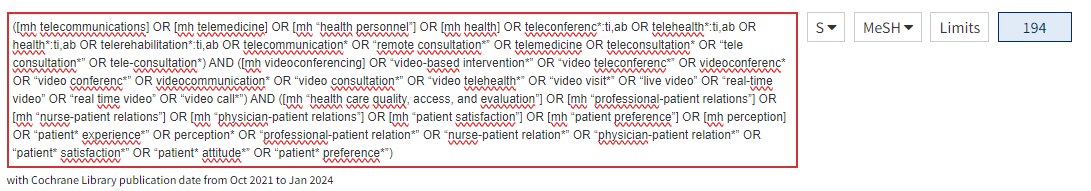

Supplement: sj-docx-2-dhj-10.1177_20552076251404513 - Supplemental material for Patients’ experiences of video consultations: A qualitative systematic review [file sj-docx-2-dhj-10.1177_20552076251404513.docx]
