# Supplementary material for: Patients’ experiences of video consultations: A qualitative systematic review
Source: Digit Health. 2026 Jan 5;12:20552076251404513. doi: 10.1177/20552076251404513 (PMC12775311; doi:10.1177/20552076251404513)
Supplement: sj-docx-3-dhj-10.1177_20552076251404513 - Supplemental material for Patients’ experiences of video consultations: A qualitative systematic review [file sj-docx-3-dhj-10.1177_20552076251404513.docx]

**Appendix 3.** Level of confidence according to CERQual approach.

| Summarised review finding | Confidence | Methodological limitations | Coherence | Adequacy | Relevance | Studies contributing to the review finding |
| --- | --- | --- | --- | --- | --- | --- |
| Main theme 1: Suitable for less complex issues when technical problems can be solved | | | | | | |
| Theme 1. Less time, effort, and travel, and more accessible. Patients feel that VC saves time, effort, energy, and trouble. Worth a lot for the persons not feeling unwell. Easy access also led to feelings of security, control, less anxiety and stress. Not having to arrange for travels was associated with a lot of benefits. | High confidence | No/Very minor concerns regarding methodological limitations | No/Very minor concerns regarding coherence | No/Very minor concerns regarding adequacy. | No/Very minor concerns regarding relevance regarding relevance because the context in our review question is health care and the data supporting this theme is only from healthcare. Our population are patients, and we have only extracted data where we have been able to the identify the patient’s perspective | Andrews et al. 2023; Chan et al. 2020; Christensen et al. 2020; Granberg et al. 2021; Grīnfelde 2022; Koppel et al. 2022; Lawson et al. 2022; Mathar et al. 2015; Moeller et al. 2022; Nissen & Lindhardt 2017; Nordtug et al. 2021; Parkinson et al. 2021; Shulver et al. 2016 |
| Theme 2. Shorter waiting times for booking appointments. Patients feel that VC was preferred because it meant less waiting when receiving bad or important news. | Moderate confidence | Moderate concerns regarding methodological limitations because only 1 ref of 3 had high reliability. 2 had unclear recruitment strategies. Nothing written on relationship | Minor concerns regarding coherence because there are only a few codes supporting this and it’s not clear why some patients had a feeling of less waiting with VC. | Moderate concerns regarding adequacy due to the number of studies and the data is not rich in detail regarding circumstances and context. | No/very minor concerns regarding relevance because the supporting data origins from healthcare only. Data was only collected when patient perspectives were identifiable. 4 of 13 studies contribute to this theme, all from different healthcare contexts. | Andrews et al. 2023; Chan et al. 2020; Granberg et al. 2021; Grīnfelde 2022; Nordtug et al. 2021 |
| Theme 3. VC for shorter, less complicated issues. Patient experience VC as shorter which suits brief follow-ups. It was meaningful time spent. VC is not a good option in acute situations or to assess complications, treatment decisions, or sensitive subjects. | High confidence | Moderate concerns regarding methodological limitations because all studies have low or moderate reliability except one (Mathar). Recruitment strategy was unknown or performed by provider, researcher and relations towards participants was not stated. | Minor concerns because many experience VC as more appropriate for follow-up and short visits, less for acute situations. | No/very minor concerns regarding adequacy because most data support this theme. | No/very minor concerns regarding relevance because the supporting data origins from healthcare only. Data was only collected when patient perspectives were identifiable. | Andrews et al. 2023; Chan et al. 2020; Christensen et al. 2020; Granberg et al. 2021; Grīnfelde 2022; Koppel et al. 2022; Mathar et al. 2015; Moeller et al. 2022; Nordtug et al. 2021; Parkinson et al. 2021 |
| Theme 4. Overcoming technical problems and discomfort. Patients change their attitude after trying VC. Acceptance came with familiarity but for some, help and support was a must. Technical issues led to feelings of frustration, mistrust, incompetence and concerns | High confidence | Minor concerns regarding methodological limitations because of lacking details on recruitment and researcher-participant relations. This was however assessed as having only minor implications on this theme. | No/very minor concerns regarding coherence. Important to remember the individuals’ different prerequisites, some learn and some needs help. | No/very minor concerns regarding adequacy because most data support this theme and are rich in details. | No/very minor concerns regarding relevance because the supporting data origins from healthcare only. Data was only collected when patient perspectives were identifiable. | Chan et al. 2020; Christensen et al. 2020; Granberg et al. 2021; Grīnfelde 2022; Koppel et al. 2022; Lawson et al. 2022; Nordtug et al. 2021; Shulver et al. 2016 |
| Main theme 2. Feeling secure, relaxed, and having mutual focus in an equitable partnership | | | | | | |
| Theme 5. A sense of privacy and security at home. Patients feel that VC offers security and privacy in the comfort of home which is appreciated and contributes to feeling calm and empowered. | High confidence | Minor concerns regarding methodological limitations because half of the studies had low or moderate reliability, and the other half high reliability. | Minor concerns regarding coherence because a considerable amount data supports this theme. There is, however, also data suggesting that certain conditions must be in place for patients to feel secure with VC at home e.g. support from family, possibility to contact at any time, the feeling of safety at home. | No/very minor concerns regarding adequacy because most data support this theme. | No/very minor concerns regarding relevance because the supporting data origins from healthcare only. Data was only collected when patient perspectives were identifiable. | Chan et al. 2020; Christensen et al. 2020; Granberg et al. 2021; Grīnfelde 2022; Mathar et al. 2015; Nissen & Lindhardt 2017; Nordtug et al. 2021; Shulver et al. 2016 |
| Theme 6. Having more time, relaxation, and focus. Patients feel relaxed in VC. Conversations are concentrated and focused. | Moderate confidence | Moderate concerns regarding methodological limitations because of lacking details on recruitment and researcher-participant relations in most studies. 2 studies had high reliability. | Minor concerns regarding coherence because most of the data support the theme. However, there is also data suggesting that VC meetings were experienced as shorter and with forced tempo. There is also contradictive data where patients felt relaxed and had more time available. | Minor concerns regarding adequacy because of lack of supporting data. Suggests that time is context dependent. | No/very minor concerns regarding relevance because the supporting data origins from healthcare only. Data was only collected when patient perspectives were identifiable. | Andrews et al. 2023; Christensen et al. 2020; Granberg et al. 2021; Grīnfelde 2022; Koppel et al. 2022; Nordtug et al. 2021; Shulver et al. 2016 |
| Theme 7. Feeling on the same level. Patients feel self-determinate and responsible in their care while being more equal to the healthcare professionals and in co-planning. | Moderate confidence | Minor concerns regarding methodological limitations because 2 of the studies have high reliability, one moderate and one low. Lacking details on recruitment and researcher-participant relations. | No/Very minor concerns regarding coherence. | Moderate concerns regarding adequacy because few studies contribute with supporting data. | No/very minor concerns regarding relevance because the supporting data origins from healthcare only. Data was only collected when patient perspectives were identifiable. 4 studies contributed to this theme, all from different healthcare contexts. | Andrews et al. 2023; Christensen et al. 2020; Granberg et al. 2021; Koppel et al. 2022; Nissen & Lindhardt 2017 |
| Theme 8. Positive to be able to see healthcare professionals. Patients appreciate to see the healthcare professionals. It improves the communication and led to control and relational benefits. | High confidence | Minor concerns regarding methodological limitations because some of the studies have low reliability and lack details on recruitment and researcher-participant relations. However, 10 of 13 studies contributed data for this theme. | No/very minor concerns regarding coherence. Remember that some found it negative to see themselves on camera. | No/very minor concerns regarding adequacy because most of the data supported this theme. | No/very minor concerns regarding relevance because the supporting data origins from healthcare only. Data was only collected when patient perspectives were identifiable | Andrews et al. 2023; Chan et al. 2020; Christensen et al. 2020; Granberg et al. 2021; Grīnfelde 2022; Koppel et al. 2022; Lawson et al. 2022; Mathar et al. 2015; Moeller et al. 2022; Nissen & Lindhardt 2017; Nordtug et al. 2021; Parkinson et al. 2021 |
| Theme 9. Needs are met similarly to FTF. Patients is satisfied with VC, would choose it again and recommend it, even to examine visual symptoms.VC is found personal and no different than FTF. | Moderate confidence | Moderate concerns regarding methodological limitations because most of the studies were assessed as having moderate reliability. | Minor concerns regarding coherence because a lot of data supports the theme, however, there are also contradictory data where patients expressed experiences pointing in opposite directions. | No/Very minor concerns regarding adequacy. | No/very minor concerns regarding relevance because the supporting data origins from healthcare only. Data was only collected when patient perspectives were identifiable. | Andrews et al. 2023; Chan et al. 2020; Christensen et al. 2020; Granberg et al. 2021; Grīnfelde 2022; Koppel et al. 2022; Lawson et al. 2022; Mathar et al. 2015; Moeller et al. 2022; Nissen & Lindhardt 2017; Nordtug et al. 2021; Shulver et al. 2016 |
| Main theme 3. Limitations regarding personal needs and practical help | | | | | | |
| Theme 10. Does not add anything and is insufficient. VC is not enough and a disadvantage for complex needs. | High confidence | Minor concerns regarding methodological limitations because the studies have high or moderate reliability but are only 5. | No/very minor concerns regarding coherence because this seem to be true for patients with a lot of knowledge about their own disease and when VC is booked "just because" without a need for it. | Minor concerns regarding adequacy because of the quantity of the data. | No/very minor concerns regarding relevance because the supporting data origins from healthcare only. Data was only collected when patient perspectives were identifiable. | Christensen et al. 2020; Granberg et al. 2021; Mathar et al. 2015; Moeller et al. 2022; Parkinson et al. 2021; Shulver et al. 2016 |
| Theme 11. More difficult to talk and ask questions. It is difficult to go into complex issues, ask questions, and open up. Disturbing technical problems prevent emotional conversations. Not being able to call whenever needed is a drawback. | High confidence | Minor concerns regarding methodological limitations because of the contributing studies to this theme, 3 studies have moderate reliability and 2 have high reliability. | No/very minor concerns regarding coherence because this is true for some patient while others experience it as easier to talk over VC. There is a theme saying that VC is only suitable for shorter, less complicated issues which also strengthen this theme. | Minor concerns regarding adequacy because although only a few studies support the theme, this is likely to be an experience that some patients have. | No/Very minor concerns regarding relevance. | Andrews et al. 2023; Christensen et al. 2020; Granberg et al. 2021; Grīnfelde 2022; Koppel et al. 2022; Moeller et al. 2022; Nissen & Lindhardt 2017; Shulver et al. 2016 |
| Theme 12. Physical exam and practical help are limited. It is negative that physical exams and practical help with paperwork and information material is limited since it is important. To consult others and get translation service is not possible in VC. | High confidence | Minor concerns regarding methodological limitations because of variations in quality. Half of the studies had high reliability, one moderate and one low. Lacking details on recruitment and researcher-participant relations. | No/Very minor concerns regarding coherence. | Minor concerns regarding adequacy because of the large quantity of data. Concerns that this theme might be pruned to be diagnose specific. | No/very minor concerns regarding relevance because the supporting data origins from healthcare only. Data was only collected when patient perspectives were identifiable. | Chan et al. 2020; Granberg et al. 2021; Grīnfelde 2022; Lawson et al. 2022; Moeller et al. 2022; Nissen & Lindhardt 2017; Shulver et al. 2016 |
| Main theme 4. Increased vulnerability and lack of emotional feedback | | | | | | |
| Theme 13. Challenges to the patient- healthcare professional relationship. It is beneficial to have an established relationship before VC and to meet first FTF. A relationship only on VC is not enough and patients prefer FTF consultations over VC. | High confidence | Minor concerns regarding methodological limitations because only half of the studies have high reliability. | No/very minor concerns regarding coherence because there is substantial data supporting the patient experience expressed in this theme. | No/Very minor concerns regarding adequacy. | No/very minor concerns regarding relevance because the supporting data origins from healthcare only. Data was only collected when patient perspectives were identifiable. | Andrews et al. 2023; Chan et al. 2020; Christensen et al. 2020; Granberg et al. 2021; Grīnfelde 2022; Koppel et al. 2022; Lawson et al. 2022; Mathar et al. 2015; Moeller et al. 2022; Nissen & Lindhardt 2017; Nordtug et al. 2021; Parkinson et al. 2021; Shulver et al. 2016 |
| Theme 14. Patients are more vulnerable at home. A good support system, like family, is crucial and patients feel more vulnerable at home. Home is a private space, and some did not like to see themselves on camera. | High confidence | Moderate concerns regarding methodological limitations because of variations in quality. Two studies were assessed as having low reliability, two with high reliability and the rest with moderate reliability. | No/very minor concerns regarding coherence because the patients are more vulnerable at home, because it’s revealing more about their personal life. But on the other hand, the security of being at home can also be experienced. This is two-folded. | Minor concerns regarding adequacy because there is substantial data supporting this theme. | No/very minor concerns regarding relevance because the supporting data origins from healthcare only. Data was only collected when patient perspectives were identifiable. | Andrews et al. 2023; Granberg et al. 2021; Koppel et al. 2022; Mathar et al. 2015; Moeller et al. 2022; Nordtug et al. 2021; Parkinson et al. 2021 |
| Theme 15. Less personal and provides less emotional support. VC is less personal, and some feel unimportant, rushed, just like the next person in line. To interpret reactions is difficult and to feel compassion, comfort, and reassurance. Patients feel insecure, distant, worried and anxious during VC. | High confidence | Minor concerns regarding methodological limitations because of lacking details on recruitment and researcher-participant relations. Two studies with high reliability. Missing information on recruitment strategies was assessed as having low impact on this particular theme | No/Very minor concerns regarding coherence. | No/Very minor concerns regarding adequacy because there is substantial data supporting this theme. | No/very minor concerns regarding relevance because the supporting data origins from healthcare only. Data was only collected when patient perspectives were identifiable. | Andrews et al. 2023; Chan et al. 2020; Christensen et al. 2020; Granberg et al. 2021; Koppel et al. 2022; Lawson et al. 2022; Moeller et al. 2022; Parkinson et al. 2021; Shulver et al. 2016; |
| Theme 16. Creates obligations and pressure. VC interfere with daily life which is disturbing and to feel stuck in front of the screen at home is an unwanted obligation and a tiresome customary routine. | Moderate confidence | Minor concerns regarding methodological limitations because of variations in quality. 3 of the studies have high reliability. | Minor concerns regarding coherence. Data shows the importance of VC supporting a specific purpose and it should not to be implemented without a reason or need. There is also contradictive data supporting the opposite, patients experiencing VC as relaxing. | Minor concerns regarding adequacy because only a few studies support this data. | No/Very minor concerns regarding relevance. | Andrews et al. 2023; Grīnfelde 2022; Koppel et al. 2022; Mathar et al. 2015; Moeller et al. 2022; Shulver et al. 2016 |
