# Supplementary material for: Patients’ experiences of video consultations: A qualitative systematic review
Source: Digit Health. 2026 Jan 5;12:20552076251404513. doi: 10.1177/20552076251404513 (PMC12775311; doi:10.1177/20552076251404513)
Supplement: sj-docx-5-dhj-10.1177_20552076251404513 - Supplemental material for Patients’ experiences of video consultations: A qualitative systematic review [file sj-docx-5-dhj-10.1177_20552076251404513.docx]

**Supplementary file 2. ENTREQ Checklist**

| No. | Item | Page Number  (where item is addressed) |
| --- | --- | --- |
| 1 | Aim | p. 2 |
| 2 | Synthesis methodology | p. 4 |
| 3 | Approach to searching | p. 2-3 |
| 4 | Inclusion criteria | p. 3 |
| 5 | Data sources | p. 2 |
| 6 | Electronic Search strategy | p. 2 & Appendix 2. |
| 7 | Study screening methods | p. 3 |
| 8 | Study characteristics | p. 4-7 |
| 9 | Study selection results | p. 5-7 |
| 10 | Rationale for appraisal | p. 4 & Appendix 3 |
| 11 | Appraisal items | p. 4 & Appendix 3 |
| 12 | Appraisal process | p. 4 |
| 13 | Appraisal results | p. 4 & Appendix 3 |
| 14 | Data extraction | p. 3-4 |
| 15 | Software | p. 3-4 |
| 16 | Number of reviewers | p. 3-4 |
| 17 | Coding | p. 4 |
| 18 | Study comparison | p. 4 |
| 19 | Derivation of themes | p. 4 |
| 20 | Quotations | p. 4-12 |
| 21 | Synthesis output | p. 14 |
